# Phylodynamics on local sexual contact networks

**DOI:** 10.1371/journal.pcbi.1005448

**Published:** 2017-03-28

**Authors:** David A. Rasmussen, Roger Kouyos, Huldrych F. Günthard, Tanja Stadler

**Affiliations:** 1 Department of Biosystems Science and Engineering, ETH Zürich, Basel, Switzerland; 2 Swiss Institute of Bioinformatics, Lausanne, Switzerland; 3 Division of Infectious Diseases and Hospital Epidemiology, University Hospital Zürich, University of Zürich, Zürich, Switzerland; 4 Institute of Medical Virology, University of Zürich, Zürich, Switzerland; CNRS, FRANCE

## Abstract

Phylodynamic models are widely used in infectious disease epidemiology to infer the dynamics and structure of pathogen populations. However, these models generally assume that individual hosts contact one another at random, ignoring the fact that many pathogens spread through highly structured contact networks. We present a new framework for phylodynamics on local contact networks based on pairwise epidemiological models that track the status of pairs of nodes in the network rather than just individuals. Shifting our focus from individuals to pairs leads naturally to coalescent models that describe how lineages move through networks and the rate at which lineages coalesce. These pairwise coalescent models not only consider how network structure directly shapes pathogen phylogenies, but also how the relationship between phylogenies and contact networks changes depending on epidemic dynamics and the fraction of infected hosts sampled. By considering pathogen phylogenies in a probabilistic framework, these coalescent models can also be used to estimate the statistical properties of contact networks directly from phylogenies using likelihood-based inference. We use this framework to explore how much information phylogenies retain about the underlying structure of contact networks and to infer the structure of a sexual contact network underlying a large HIV-1 sub-epidemic in Switzerland.

## Author summary

Phylodynamic models relate the branching pattern of a pathogen’s phylogenetic tree to the tree-like growth of an epidemic as it spreads through a host population. Such models are increasingly used to learn about the epidemiology of different pathogens. We extend current models to consider the structure of host contact networks—the web of physical interactions through which pathogens spread. By considering how local interactions among hosts shape the phylogeny of a pathogen, our models offer a “pathogen’s eye view” of these networks. Our models also provide a statistical framework that can be used to infer network structure directly from phylogenies, which we use to estimate the properties of a sexual contact network in Switzerland from a HIV phylogeny.

## Introduction

From the viewpoint of an infectious pathogen, host populations are highly structured by the physical contacts necessary for disease transmission to occur. For pathogens whose transmission does not require intimate or sustained physical contact, random mixing models assuming contacts form instantaneously between individuals may offer a reasonable approximation to the true dynamics of person-to-person contact [1–3]. But for pathogens like sexually-transmitted infections (STIs), transmission requires contacts that are generally more limited in number, less transient in nature, and form non-randomly based on individual behavior—resulting in host populations that are highly structured locally at the level of individuals [4–7]. Even for non-STIs, a limited number or clustering among contacts may constrain transmission; potentially explaining why newly emerging infections like SARS and Ebola give rise to large outbreaks in some social settings but not others [8–10]. It is therefore often more reasonable to view communities as networks of individuals connected by edges that represent the physical contacts through which transmission can occur. Through the study of theoretical network models, epidemiologists now understand that contact network structure has a profound influence on epidemic dynamics and whether or not control strategies will be effective [8, 11–14]. Yet studying the structure of contact networks empirically through methods such as contact tracing is difficult and costly, meaning we often know little about the structure of contact networks underlying real-world epidemics [15, 16].

New hope for the empirical study of contact networks has emerged in recent years from the widespread availability of pathogen molecular sequence data. In molecular epidemiology, sequence data is already commonly used to link individuals into probable transmission pairs or clusters based on the phylogenetic distances between their pathogens. While such approaches do not directly reveal the structure of contact networks, they can reveal paths in the contact network through which the pathogen spread and provide a useful heuristic for assessing how well connected networks are within and between different subpopulations or risk-groups [17–19]. Other methods in molecular epidemiology attempt to reconstruct the full details of the underlying transmission tree, the directed graph showing exactly who infected whom in an outbreak [20–25]. Essentially though, all current methods for inferring linkage and transmission trees take a bottom-up approach—they attempt to reconstruct routes of transmission by linking sampled individuals based on their phylogenetic distance. While this can be a powerful approach for studying densely sampled outbreaks where most infected individuals are sampled, bottom-up approaches may provide misleading results when applied to sparsely sampled epidemics. In this case, two infected individuals may have pathogens that are most closely related to one another phylogenetically but an unknown number of intervening infections might separate them in the true transmission tree. Thus the phylogenetic proximity of individuals may only weakly correlate with their proximity in the transmission tree, making it very difficult to reconstruct the detailed transmission history of who infected whom.

While it may not be possible to reconstruct the detailed structure of transmission networks from sparsely sampled data, it may still be possible to infer large-scale properties of contact networks. By simulating the phylogenetic history of pathogens spreading through networks, recent studies have shown that network properties can exert a strong influence on the structure of phylogenetic trees [26–28]. For example, increasing levels of contact heterogeneity—variation in the number of contacts individuals form—can result in increasingly asymmetric or imbalanced trees and shift the distribution of coalescent (i.e. branching) events earlier towards the beginning of an epidemic [26, 27]. However, statistical measures of tree topology like imbalance may only weakly correlate with network statistics like contact heterogeneity, and may also be highly dependent on how samples are collected [28]. Moreover, in addition to network structure, population dynamics also strongly shape phylogenies and therefore potentially confound inferences of network structure drawn from phylogenies [28]. For example, clustering of samples together in phylogenetic trees has previously been assumed to indicate clustering of individuals in the underlying contact network, but phylogenetic clustering can arise naturally in epidemics even when no measurable degree of clustering exists in a population [29]. Taken together then, previous work suggests that contact network structure can shape pathogen phylogenies, but we do not yet know how to properly extract this information from trees.

In this paper, we present a new theoretical framework for relating pathogen phylogenies to contact networks using phylodynamic modeling. Our approach is quite different from bottom-up approaches in that it does not attempt to reconstruct the details of person-to-person transmission. Rather, we start with a random graph model [30] that captures the important statistical properties of real-world networks. We then use pairwise epidemic models [31–33] to capture the population dynamics of an epidemic on a network with the statistical properties specified by the random graph model. In addition to tracking the infection status of individuals, these pairwise models track the status of pairs of individuals and thereby correlations in the infection status of neighboring individuals, such as the depletion of susceptible hosts around infected individuals. Analogously, by shifting our focus from the level of individuals to the level of pairs, we derive a relatively simple coalescent model that captures a pathogen’s phylogenetic history as a backwards-time dynamical process on a network. The pairwise coalescent model naturally takes into account incomplete sampling and how network structure and epidemic dynamics interact to shape pathogen phylogenies. By considering phylogenies in a probabilistic framework, the pairwise coalescent model also allows us to compute the likelihood of a given phylogeny evolving on a network with defined statistical properties, and therefore to estimate the structure of networks from phylogenies using likelihood-based inference.

How local contact network structure shapes pathogen phylogenies has received some attention in recent years [26–28], but has not been comprehensively studied. After deriving the pairwise coalescent model, we therefore begin by using simulations to explore how network properties such as overall connectivity, clustering, contact heterogeneity and assortativity shape phylogenies. Using these simulations, we demonstrate that the pairwise coalescent model captures how these network properties shape phylogenies in terms of coalescent times, how lineages move through a network, and overall tree topology. We then go on to show that the model can be used to accurately estimate network properties from phylogenies, although how precisely depends strongly on sampling effort. Finally, we have implemented the model in BEAST 2 [34] as a package called PairTree, which we use to estimate the structure of a contact network underlying a large HIV sub-epidemic among men-who-have-sex-with-men (MSM) in Switzerland.

## Models and methods

Our phylodynamic modeling framework is composed of three interacting components. The first two components, random graph and pairwise epidemic models, are well described in the literature and we only briefly review the necessary concepts and notation here. Instead, we focus on the third and novel component of our framework, the pairwise coalescent model, which we derive from the pairwise epidemic model.

### Random graph models

In network epidemiology, random graph models are often used to model the large-scale statistical properties of networks while treating the fine-scale details of who is connected to whom as random. Random graph models can therefore be thought of as a probability distribution on graphs constrained to take on certain statistical properties. Here, we use the configuration model [35] and extensions thereof to model network structure and generate random graphs parameterized to vary in overall connectivity, clustering, contact heterogeneity and assortative mixing.

#### A. Connectivity

Connectivity quantifies how well-connected individual nodes are in a network in terms of their degree, or number of contacts. In the simplest case of the configuration model, all *N* nodes are assigned a fixed degree $\hat{k}$ and then randomly connected to other nodes through edges, resulting in a homogenous or *k*-regular random graph. The parameter $\hat{k}$ therefore quantifies the overall connectivity of the network.

#### B. Clustering

Clustering is defined as the probability that two nodes connected to a common neighbor are also connected to one another, and therefore quantifies how locally interconnected networks are [31]. Clustering can be quantified in terms of a clustering coefficient *ϕ*:

$$\begin{array}{c} \phi =\frac{3\times \text{number}\;\text{of}\;\text{triangles}}{\text{number}\;\text{of}\;\text{connected}\;\text{triples}}, \end{array}$$ (1)

where a triangle refers to a closed loop of three connected nodes and a triple to three linearly connected nodes [30].

To introduce clustering into random networks, we use the triangular configuration model [36, 37]. Under this model, rather than defining the degree distribution *d*_*k*_, we define a joint degree distribution *d*_*st*_ on the probability that a node is connected to *s* neighbors not forming triangles and 2*t* other neighbors through triangles, and thus has total degree *k* = *s* + 2*t*. As shown by [37], given *d*_*st*_ and the overall degree distribution *d*_*k*_, the expected clustering coefficient is

E(ϕ)=∑s,ttdst∑kk2dk. (2)

#### C. Contact heterogeneity

Contact heterogeneity refers to variation in the number of contacts individuals form in a network and can be quantified by the variance $\sigma_{k}^{2}$ in the degree distribution *d*_*k*_. Networks with any arbitrary degree distribution can be generated under the standard configuration model by assigning each node *n* = 1, 2, …, *N* a degree according to a random degree sequence *k*_1_, *k*_2_, …, *k*_*N*_ drawn from *d*_*k*_. Each individual node *n* is then randomly joined to *k*_*n*_ other nodes to form the edges of the network.

#### D. Assortative mixing

Assortative mixing is the tendency for individuals to form connections with individuals similar to themselves, leading to correlations between the properties of adjacent nodes in a network [30]. Here, we introduce correlations in the degree of connected nodes by specifying the edge degree distribution *e*_*kl*_, which gives the probability of a randomly chosen edge connecting a degree *k* to a degree *l* node. The strength of assortative mixing can be quantified in terms of the assortativity coefficient *r* given *e*_*kl*_ and *d*_*k*_:

$$\begin{array}{c} r=\frac{\sum_{k}\sum_{l}kl\left(e_{kl}-d_{k}d_{l}\right)}{\sigma_{k}^{2}}. \end{array}$$ (3)

Thus, *r* can also be interpreted as the Pearson correlation coefficient in the degree of nodes connected by edges in the network.

To get a one-parameter random graph model that allows for the strength of assortative mixing to vary based on *r*, we follow [38] and constrain each entry in *e*_*kl*_ to follow the form

$$\begin{array}{c} e_{kl}=d_{k}d_{l}+r\sigma_{k}^{2}\left(d_{k}-x_{k}\right)\left(d_{l}-x_{l}\right), \end{array}$$ (4)

where *x*_*k*_ is a normalized distribution chosen such that *e*_*kl*_ is never negative. To our knowledge, there is no algorithm that allows for direct simulation of networks from the distribution over graphs defined by *e*_*kl*_. We therefore sample networks using the Metropolis-Hastings sampler also proposed by [38] that iteratively rewires networks until convergence on a target distribution defined by *e*_*kl*_ is reached.

### Pairwise epidemic model

The second component of our modeling framework consists of epidemiological models that describe the dynamics of a pathogen spreading through a network with statistical properties specified by a random graph model. As in standard SIR-type epidemiological models, we track the infection status of each host node as susceptible or infected, along with an optional recovered class. We use the notation [*S*_*k*_] and [*I*_*k*_] to denote the number of degree *k* susceptible and infected individuals. [*S*_*k*_*I*_*l*_] denotes the the number of pairs in the network connecting susceptible individuals with degree *k* to infected individuals with degree *l*. At the level of individuals, the epidemic dynamics are described by the following differential equations:

$$\begin{array}{ccc} \frac{d[S_{k}]}{dt} & = & -\tau \sum_{l}\left[S_{k}I_{l}\right]+\delta_{0,1}\nu \left[I_{k}\right]\\ \frac{d[I_{k}]}{dt} & = & \tau \sum_{l}\left[S_{k}I_{l}\right]-\nu \left[I_{k}\right] \end{array}$$ (5)

Here, *τ* is the per-contact rate at which infected individuals transmit to their neighbors and *ν* is the removal or recovery rate. The dummy variable *δ*_0,1_ is set to either 1 or 0 depending on if recovered individuals become susceptible again (the SIS model) or are immunized (the SIR model).

As seen from Eq (5), the transmission dynamics depend on the [*S*_*k*_*I*_*l*_] terms and thus how individuals are connected into pairs or partnerships. We therefore need to track the dynamics at the level of pairs, which in turn depends on the number of triples such as [*S*_*k*_*S*_*l*_ *I*_*m*_] where *m* is the degree of the third node:

$$\begin{array}{ccc} \frac{d[S_{k}S_{l}]}{dt} & = & -\tau \sum_{m}\left(\left[S_{k}S_{l}I_{m}\right]+\left[I_{m}S_{k}S_{l}\right]\right)+\delta_{0,1}\nu \left(\left[S_{k}I_{l}\right]+\left[I_{k}S_{l}\right]\right)\\ \frac{d[S_{k}I_{l}]}{dt} & = & \tau \sum_{m}\left(\left[S_{k}S_{l}I_{m}\right]-\left[I_{m}S_{k}I_{l}\right]\right)-\tau \left[S_{k}I_{l}\right]-\nu \left[S_{k}I_{l}\right]+\delta_{0,1}\nu \left[I_{k}I_{l}\right]\\ \frac{d[I_{k}I_{l}]}{dt} & = & \tau \sum_{m}\left(\left[I_{k}S_{l}I_{m}\right]+\left[I_{m}S_{k}I_{l}\right]\right)+\tau \left(\left[S_{k}I_{l}\right]+\left[I_{k}S_{l}\right]\right)-2\nu \left[I_{k}I_{l}\right] \end{array}$$ (6)

These are the pairwise network equations introduced by [31] and extended to heterogenous contact networks by [33]. By tracking the status of pairs rather than just individuals, the pairwise equations take into account local correlations that build up over time between the infection status of neighboring nodes; hence their other common name, correlation equations [32]. These local correlations arise because a node’s infection status depends strongly on the status of its neighbors. For example, early on in an epidemic positive correlations develop between infected individuals, reflecting the fact that infected individuals are likely to be surrounded by other infected individuals who either infected them or became infected by them. Because these correlations can have a strong impact on epidemic dynamics, such as through the local depletion of susceptible nodes surrounding infected nodes, tracking these correlations allows pairwise models to more accurately describe epidemic dynamics on networks. The initial conditions for the pairwise epidemic model depend on the degree distribution *d*_*k*_ and edge degree distribution *e*_*kl*_ of the network and are described in S1 Text.

While the dynamics at the level of pairs depends on the number of triples, which in turn depends on even higher-order configurations, previous work has shown that moment closure methods can be used to approximate the number of triples based on the number of pairs without much loss of accuracy [33, 39]. We thus “close” the system at the level of pairs by approximating each triple of arbitrary type [*ABC*] as:

$$\begin{array}{c} \left[ABC\right]=\frac{l-1}{l}\frac{\left[AB][BC\right]}{\left[B\right]}\left(\left(1-\phi \right)+\frac{N}{l}\frac{\left[AC\right]}{\left[A][C\right]}\phi \right), \end{array}$$ (7)

where *l* is the degree of the central node in state *B*. By taking into account the clustering coefficient *ϕ*, this moment closure takes into account additional state correlations that can arise between three nodes when there is appreciable clustering in the network [31, 32].

The basic reproductive number *R*_0_, as usually defined, is the average number of secondary cases resulting from a single infectious individual in an otherwise susceptible population. As shown in [33], for the pairwise model on a heterogenous network,

$$\begin{array}{c} R_{0}=\frac{\tau (\lambda_{max}-1)}{\nu }, \end{array}$$ (8)

where λ_*max*_ is the dominant eigenvalue of the next generation matrix *M*, which has elements

$$\begin{array}{c} M_{kl}=\frac{\left(l-1\right)\left[N_{k}N_{l}\right]}{l[N_{l}]}. \end{array}$$ (9)

Here, *N*_*k*_ and *N*_*l*_ represent the total number of individuals in the network with degree *k* and *l*.

### Pairwise coalescent model

The third and novel component of our modeling framework are coalescent models that allow us to probabilistically relate the phylogenetic history of a pathogen back to the dynamics of an epidemic on a network. In essence, these coalescent models provide a probability distribution over trees, and therefore allow us to compute the likelihood of a given phylogeny having evolved on a random graph with known statistical properties. While coalescent theory has previously been extended to accommodate the nonlinear transmission dynamics of infectious pathogens [40–43], these coalescent models assume random mixing, at least within discrete subpopulations, and therefore neglect local contact network structure. Below, we extend the structured coalescent framework of [43] to include local contact network structure by shifting our focus from the level of individuals to pairs of hosts in the network.

The likelihood of a time-scaled phylogeny T under a structured coalescent model with parameters *θ* has the general form:

$$\begin{array}{c} L\left(T|\theta \right)=\prod_{p=1}^{P-1}\lambda_{ij}\left(t_{p}\right)\exp\left[-\int_{s=t_{p}}^{s=t_{p+1}}\sum_{i}^{i\in A\left(s\right)}\sum_{j>i}^{j\in A\left(s\right)}\lambda_{ij}\left(s\right)ds\right]. \end{array}$$ (10)

For a tree containing *P* samples, the total likelihood is the product of the likelihood of each of the *P* − 1 coalescent events and the waiting times between events. The likelihood of each coalescent event depends on the rate λ_*ij*_(*t*_*p*_) at which lineages *i* and *j* coalesce at time *t*_*p*_. These rates may depend on the location of lineages *i* and *j*, and thus this formulation of the coalescent likelihood accommodates population structure. The likelihood of the waiting time between coalescent events is given by the exponential term in Eq (10). Here, the sums are over all lineages A(s) present in the phylogeny at time *s*, which is allowed to change within coalescent intervals due to sampling.

The pairwise coalescent rates λ_*ij*_ are centrally important to our model as they are required to compute the likelihood in Eq (10) and provide the main link between the epidemic dynamics and the coalescent process. To derive these rates, we begin by making the simplifying assumption common in phylodynamics that only a single pathogen lineage resides in each infected host. While this assumption ignores within-host pathogen diversity, it dramatically simplifies the relationship between transmission events and coalescent events in the pathogen phylogeny: each coalescent event in the phylogeny will represent a transmission event on the network. Below, we use this relationship to derive the pairwise coalescent rates λ_*ij*_ for pairs of lineages.

#### Pairwise coalescent rates

To derive the pairwise coalescent rate λ_*ij*_, we first consider the probability that lineages *i* and *j* coalesce conditional on a transmission event occurring somewhere in the network. In order for two lineages to coalesce at a transmission event from an individual with *l* contacts to an individual with *k* contacts, we can reason that at the time of the event three conditions must hold:

1. The two lineages must be in two infected individuals, one with *k* and the other with *l* contacts.
2. The two lineages must reside in two nodes connected in a *I*_*k*_*I*_*l*_ pair.
3. The two lineages must be in the specific *I*_*k*_*I*_*l*_ pair involved in the transmission event.

We note that each of these conditions must be met in turn for the remaining conditions to be met. We will therefore consider the probability that each of these conditions is true in turn conditional on the preceding conditions having been met. In this case, *Pr*(2) ≔ *Pr*(2|1) and *Pr*(3) ≔ *Pr*(3|1, 2), such that the joint probability of all three being true *Pr*(1, 2, 3) = *Pr*(1)*Pr*(2|1)*Pr*(3|1, 2).

First, consider the probability that lineages *i* and *j* are in two infected individuals; one in a *I*_*k*_ node and the other in a *I*_*l*_ node. In general, we will not know the degree of the infected node in which the lineage resides (for shorthand, we will refer to this as the lineage’s state). We must therefore treat the state of lineages probabilistically and will use the notation *p*_*ik*_ to represent the probability that lineage *i* resides in a degree *k* infected node. The probability *p*_*ik*_ will evolve over time, and we show below how we track *p*_*ik*_ backwards through time. The probability $P_{kl}$ that lineages *i* and *j* reside in nodes with degrees *k* and *l* is then equal to the probability that lineage *i* is in state *k* and lineage *j* is in state *l* or vice versa, such that

$$\begin{array}{c} P_{kl}=\left\{\begin{array}{cc} p_{ik}p_{jl}+p_{il}p_{jk}, & \text{if}\;k\neq l\\ p_{ik}p_{jl}, & \text{if}\;k=l. \end{array}\right. \end{array}$$ (11)

Second, consider the probability that lineages *i* and *j* reside in two nodes connected in a *I*_*k*_*I*_*l*_ pair. The total number of possible pairs between *I*_*k*_ and *I*_*l*_ nodes is [*I*_*k*_][*I*_*l*_] if *k* ≠ *l* or ([Ik]2) if *k* = *l*. Because pairs are assumed to form randomly under our random graph models, the probability *χ*_*kl*_ that a randomly chosen *I*_*k*_ node is connected to a random *I*_*l*_ node in a *I*_*k*_*I*_*l*_ pair is:

χkl=[IkIl][Ik][Il],ifk≠l[IkIl][Ik]2,ifk=l. (12)

Given that our two lineages reside in *I*_*k*_ and *I*_*l*_ nodes, then the probability that our lineages reside in a *I*_*k*_*I*_*l*_ pair is *χ*_*kl*_.

Third, given that lineages *i* and *j* are in two nodes that form a *I*_*k*_*I*_*l*_ pair, the probability that it is this pair out of all *I*_*k*_*I*_*l*_ pairs in the network that was involved in a given transmission event is simply 1/[*I*_*k*_*I*_*l*_].

Note that all three of these probabilities evolve over time to reflect the changing distribution of infectious contacts in the network through their dependence on the epidemic dynamics described by the pairwise epidemic model, which in turn reflects the degree distribution *d*_*k*_ and the edge degree distribution *e*_*kl*_ in the underlying random graph model. For example, in a model with assortative mixing, correlations in the degree of connected individuals as described by *e*_*kl*_ will be reflected in [*I*_*k*_*I*_*l*_], and therefore influence *χ*_*kl*_ as [*I*_*k*_*I*_*l*_] evolves over the course of an epidemic.

These three probabilities collectively give the probability that lineages *i* and *j* coalesce at a particular transmission event. Multiplying these probabilities by the total rate at which degree *l* nodes transmit to degree *k* nodes, the rate at which lineages *i* and *j* coalesce through *l* → *k* transmission events is:

$$\begin{array}{c} \lambda_{ij}^{l\to k}=\frac{\tau \left[S_{k}I_{l}\right]\chi_{kl}}{\left[I_{k}I_{l}\right]}P_{kl}. \end{array}$$ (13)

Summing over all possible transmission events with respect to the degree of the nodes involved, we arrive at the total pairwise coalescent rate:

$$\begin{array}{c} \lambda_{ij}=\sum_{k}\sum_{l}\frac{\tau \left[S_{k}I_{l}\right]\chi_{kl}}{\left[I_{k}I_{l}\right]}P_{kl}. \end{array}$$ (14)

The likelihood given in Eq (10) can then be computed by plugging in λ_*ij*_(*t*) for each lineage pair after numerically integrating the ODEs for [*S*_*k*_*I*_*l*_] and [*I*_*k*_*I*_*l*_] given in Eq (6) up to time *t*.

For a homogenous network where all nodes have the same degree $\hat{k}$, Eq (14) simplifies to

$$\begin{array}{c} \lambda_{ij}=\frac{\tau [SI]\chi }{\left[II\right]}. \end{array}$$ (15)

In a fully connected network where each node has degree $\hat{k}=N-1$, every node is connected to every other node. In this limiting case, we expect the dynamics of an epidemic on a network to be the same as under a random mixing model with a transmission rate *β* scaled so that infectious contacts occur at the same rate under both models. In this case, the probability that two random infected nodes are connected in a pair *χ* ⇒ 1, [*SI*] ⇒ *SI*, and [II]⇒(I2). Making these substitutions in Eq (15), we see that

λ=τ[SI]χ[II]⇒βSII2≈2βSI. (16)

This is the same pairwise coalescent rate derived by [40] for a random mixing SIR-type model. We therefore see that the pairwise coalescent model and earlier coalescent models assuming random mixing converge in the limit of a fully connected network.

#### Tracking lineage movement

We now consider how individual pathogen lineages move through a network. Because we need to know the probabilities *p*_*ik*_ of a lineage residing in a degree *k* host in order to compute the pairwise coalescent rates given in Eq (14), we probabilistically track the movement of lineages by tracking how *p*_*ik*_ changes backwards through time along a lineage using a framework based on master equations previously developed by [43]. These master equations have the general form

$$\begin{array}{c} \frac{d}{dt}p_{ik}=\sum_{l}\left(\gamma^{k\leftarrow l}p_{il}-\gamma^{l\leftarrow k}p_{ik}\right), \end{array}$$ (17)

where *γ*^*k*←*l*^ is the rate at which lineages transition from degree *l* to degree *k* hosts backwards in time. How these transition rates are computed and further details about how the ancestral degree distribution *p*_*k*_ is computed for each lineage in a phylogeny are described in S1 Text, where we also show that these master equations accurately describe how lineages move through networks (S1 Fig).

### Summary of the general approach

To summarize, our approach uses a random graph model to describe the statistical properties of a network including its degree distribution. These statistical properties are then used to initialize the state variables in the pairwise epidemic model—including the degree distribution of susceptible and infected individuals in the network. The ODEs for state variables like the number of [*S*_*k*_*I*_*l*_] pairs given in Eqs (5) and (6) are then solved forward in time. Given these forward-time dynamics, the lineage state probabilities can be solved backwards in time along each lineage in the phylogeny according to Eq (17). With both the epidemic state variables and lineage state probabilities at hand, the pairwise coalescent rates can be computed according to Eq (14). Finally, with these coalescent rates the likelihood of a phylogeny can be calculated using Eq (10), allowing us to infer network parameters in either a maximum likelihood (ML) or Bayesian framework. For ML inference, we use a numerical optimization routine (*fminsearch* in Matlab) to find the parameter values that maximize the likelihood of a given phylogeny. For Bayesian inference, we use a MCMC approach to sample from the posterior distribution. For the latter, the pairwise coalescent model was implemented in BEAST 2. Details on how the MCMC analysis was performed are provided in the input XML files provided along with the source code at https://github.com/davidrasm/PairTree.

## Results

### The coalescent process on random networks

To see how key network properties shape pathogen phylogenies and how well the pairwise coalescent model captures their effects, we generated networks under random graph models parameterized to obtain networks with known statistical properties. On top of these networks, we simulated the spread of an epidemic using individual-based stochastic (IBS) simulations that tracked the ancestry of each pathogen lineage forward in time so that a true phylogeny was obtained from each simulation (see S1 Text). We then compared the epidemic dynamics and phylogenies simulated under the IBS model to those expected under the pairwise epidemic and coalescent models.

**Fig 1:**
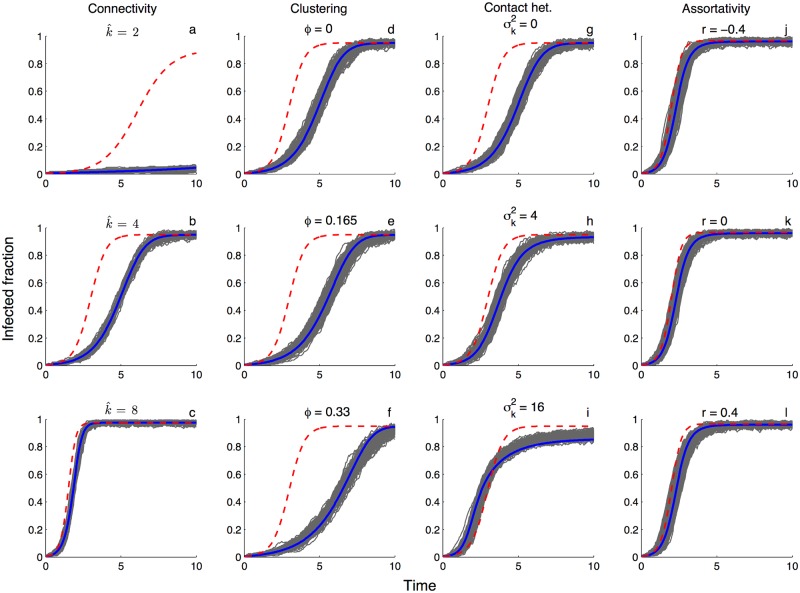
Comparison of SIS epidemic dynamics on networks with different statistical properties. Grey lines show 500 stochastic realizations of the individual-based model run on different random networks. Colored lines show the mean dynamics expected under the pairwise epidemic model (blue) versus the random mixing model (red). **(a-c)** Homogenous networks with varying overall connectivity $\hat{k}$. **(d-f)** Homogenous networks with different clustering coefficients *ϕ* but fixed degree $\hat{k}=4$. **(g-i)** Heterogenous networks with constant mean degree *μ*_*k*_ = 4 but with different variances $\sigma_{k}^{2}$ in the degree distribution. **(j-l)** Heterogenous networks with different assortativity coefficients *r*. To allow for greater assortativity, the mean and variance of the degree distribution was raised to six. For all simulations the network size *N* = 250, the transmission rate *τ* = 0.5 and the recovery rate *ν* = 0.1. The transmission rate *β* under random mixing was scaled so that the rate of infectious contacts was the same as under the pairwise epidemic model at *t* = 0, giving the two models the same intrinsic growth rate.

**Fig 2:**
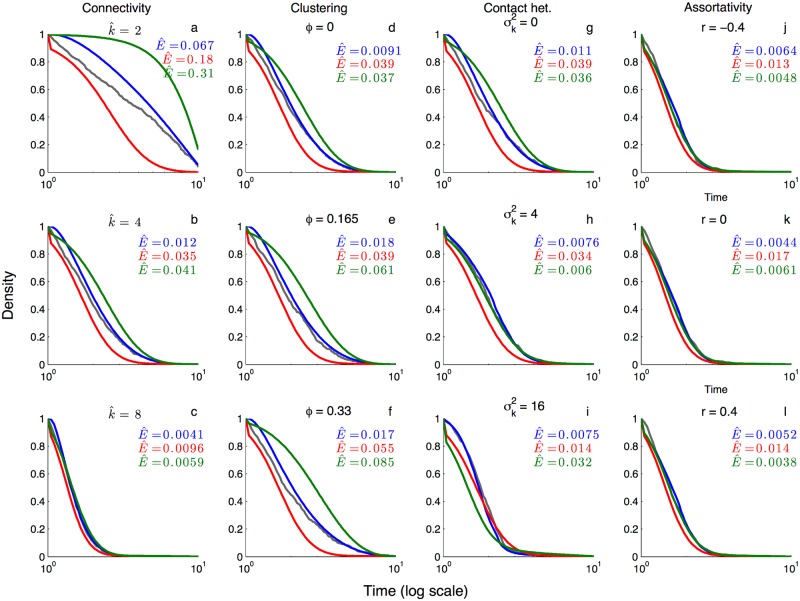
The cumulative distribution of coalescent event times for the networks and epidemic dynamics shown in Fig 1. Grey lines show the observed distribution of coalescent events obtained by tracking two randomly sampled lineages back in time until they coalesce in 500 individual-based stochastic simulations. Colored lines show the theoretically predicted coalescent densities under the pairwise coalescent (blue), the random mixing coalescent (red) and the random mixing coalescent conditioned on the mean dynamics provided by the pairwise epidemic model (green). The $\hat{E}$ values give the mean time-integrated error in the coalescent density predicted by each model relative to the stochastic simulations. **(a-c)** Homogenous networks with varying overall connectivity $\hat{k}$. **(d-f)** Homogenous networks with different clustering coefficients *ϕ*. **(g-i)** Heterogenous networks with different variances $\sigma_{k}^{2}$ in the degree distribution. **(j-l)** Heterogenous networks with different assortativity coefficients *r*. All network and epidemiological parameters are the same as in Fig 1.

As expected from earlier work [14, 39, 44], the pairwise epidemic model provides an excellent deterministic approximation to the mean dynamics observed in IBS simulations across a wide range of random networks, whereas random mixing models generally do not (Fig 1). Likewise, the pairwise coalescent model does an excellent job of capturing the coalescent process on these networks in terms of the temporal distribution of coalescent events over the epidemic (Fig 2, blue). In contrast, the coalescent distributions expected under a random mixing coalescent model provide a reasonable approximation on some networks but not others (Fig 2, red). For example, on poorly connected and highly clustered networks, the expected distribution of coalescent times under random mixing deviates widely from the IBS simulations. This is the case even if we condition the random mixing model on the more accurate population trajectories predicted by the pairwise epidemic model (Fig 2, green). On better connected networks and on networks with more contact heterogeneity, the random mixing model does almost as well as the pairwise coalescent model. In the S1 Text, we additionally explore when the pairwise approximation fails due to the presence of higher-order network structure (see S2 Fig).

### Network effects on tree topology

In addition to coalescent time distributions, contact network structure can shape the topology of phylogenies. In particular, pathogens residing in well connected hosts may have the opportunity to infect more hosts and therefore leave more descendent lineages, causing trees to become increasingly asymmetric or imbalanced as the amount of contact heterogeneity increases in a population [26–28, 45]. We therefore simulated trees on random networks with different levels of contact heterogeneity using IBS simulations and under the pairwise coalescent model using backward-time simulations in order to see if the coalescent model can capture the effects of contact heterogeneity on tree imbalance. Trees were compared using three of the most widely used measures of tree imbalance [46]: Colless’s index, Sackin’s index and the number of cherries. How these statistics were computed and normalized for trees of different sizes is described in the S1 Text.

**Fig 3:**
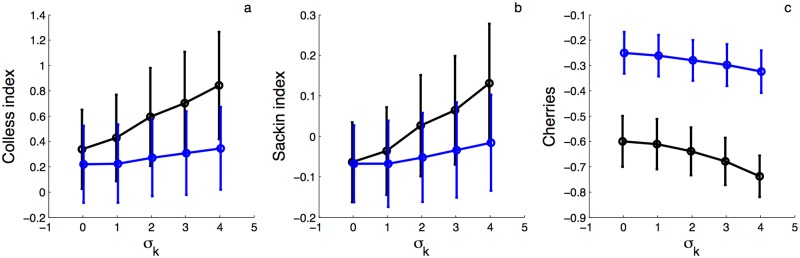
Phylogenetic tree imbalance on networks with increasing levels of contact heterogeneity. Trees were simulated using stochastic, individual-based simulations on random networks (black lines) or using backward-time simulations of the pairwise coalescent model (blue line). **(a-c)** Imbalance measured in terms of Colless’ index, Sackin’s index and the total number of cherries for trees simulated with increasing levels of contact heterogeneity. All three imbalance measures are normalized according to the expected value of the statistic for a Yule tree with the same number of samples. Circles and vertical lines mark the mean and standard deviation of the imbalance measures for 1000 simulated trees. All simulations were performed with *N* = 250 and a sampling fraction of *ρ* = 0.5.

Trees generated by IBS simulations and under the pairwise coalescent model both grow increasingly imbalanced with increasing contact heterogeneity (Fig 3). Colless’ and Sackin’s index both increase with greater contact heterogeneity, while the number of cherries decreases as expected for more imbalanced trees. However, IBS trees are more imbalanced overall than coalescent trees and grow disproportionally more so with increasing contact heterogeneity. This discrepancy may be due to additional network structure above the level of pairs not accounted for in the pairwise models—leading to additional variability in transmission potential for lineages in different parts of a network. Thus, it appears that the pairwise coalescent can partially capture the effects of local contact structure on tree topology, although the effect of network structure appears to be stronger in trees evolving on actual networks.

### Inference

The pairwise coalescent model allows us to compute the likelihood of a given phylogeny being generated by an epidemic on a random graph with defined statistical properties. It is therefore possible to estimate the statistical properties of a network directly from a phylogeny. However, phylogenies may retain little information about contact network structure, especially if the epidemic is sparsely sampled. To explore the information content of phylogenies regarding network structure, we simulated epidemics on random networks with known statistical properties. A variable fraction of infected nodes was then sampled upon removal to obtain phylogenies with sampling fractions *ρ* of 10, 25, 50 and 100%. The pairwise coalescent model was then used to construct likelihood profiles for each parameter controlling local network structure while all other epidemiological parameters were fixed at their true values.

**Fig 4:**
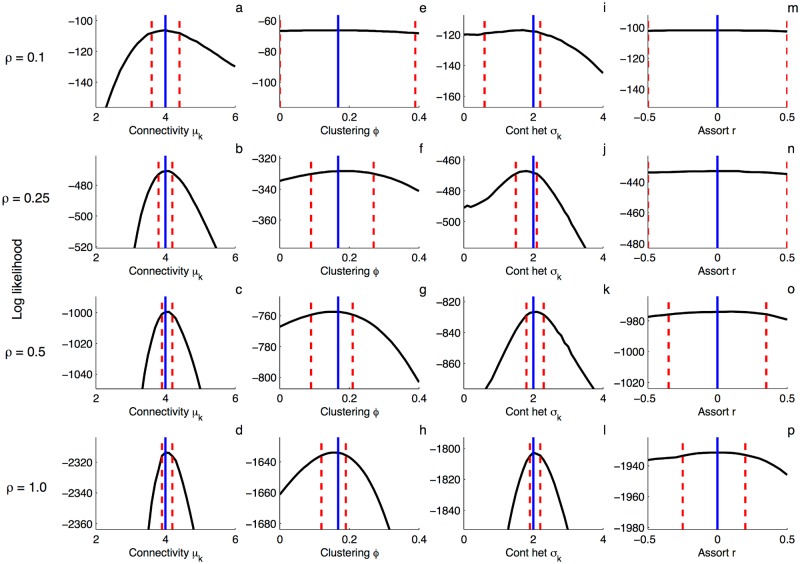
Likelihood profiles for network parameters inferred from phylogenies containing a variable fraction of all infected nodes *ρ*. Solid blue lines indicate true parameter values and dashed red lines are the approximate 95% confidence intervals derived from the quantiles of the chi-squared distribution with one degree of freedom. Parameters were estimated one at a time, with all other parameters fixed at their true values. **(a-d)** Overall connectivity as parameterized by the mean degree *μ*_*k*_. Here we assume the degree distribution follows a discretized gamma distribution with a fixed standard deviation *σ*_*k*_ = 2 **(e-h)** Clustering as parameterized by the clustering coefficient *ϕ*. **(i-l)** Contact heterogeneity as parameterized by the standard deviation of the degree distribution *σ*_*k*_. The mean of the degree distribution was fixed at *μ*_*k*_ = 4. **(m-p)** Assortativity as parameterized by the assortativity coefficient *r* of the edge degree distribution. For assortativity, the true degree of all sampled nodes was provided when computing the likelihood profiles. Trees were simulated on networks with *N* = 250 and the epidemiological parameters were fixed at the values used in Figs 1 and 2.

At sampling fractions at or below 10%, except for overall connectivity the simulated phylogenies contain little or no information about local network structure, as seen from the essentially flat likelihood profiles (Fig 4). At sampling fractions ≥ 25%, the likelihood profiles begin to show significant curvature for clustering and contact heterogeneity, and with sampling fractions ≥ 50% the likelihood profiles are sharply curved enough that these parameters can be estimated rather precisely with narrow 95% confidence intervals. Assortativity appears more difficult to infer from phylogenies, even if the true degree of sampled nodes is provided. Although the likelihood profiles for *r* do show some curvature at sampling fractions ≥ 50%, the credible intervals remain relatively wide even with complete sampling.

**Fig 5:**
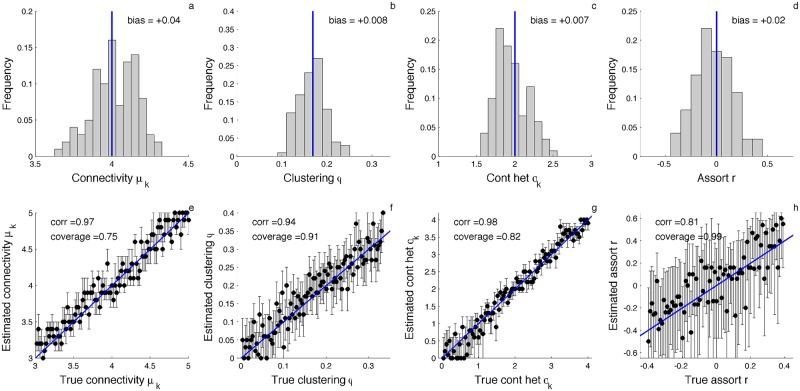
Likelihood-based estimates of network parameters and their statistical performance. **(a-d)** Distribution of maximum likelihood point estimates of the mean connectivity (degree) *μ*_*k*_, clustering coefficient *ϕ*, contact heterogeneity *σ*_*k*_ and assortativity coefficient *r* for 100 simulated phylogenies. **(e-h)** Maximum likelihood estimates (dots) and 95% confidence intervals (lines) for each network property under different model parameterizations. Blue lines indicate the true parameter value used in each simulation. Correlation (corr) refers to the Pearson correlation between the true and estimated parameter values. Coverage refers to the fraction of simulations in which the true parameter falls within the estimated 95% confidence intervals. All simulations were performed with a fraction *ρ* = 0.5 of infected individuals sampled upon removal serially over time.

To check for potential biases in our estimates of network parameters, we simulated 100 additional phylogenies under a fixed value of each parameter using forward-time IBS simulations. We then obtained a maximum likelihood estimate (MLE) of the corresponding parameter. The MLEs appear centered around the true parameter values with little to no detectable bias (Fig 5a–5d).

Next, we simulated trees under a wider range of parameter values for each network property to check how well our estimator performs under different model parameterizations. Overall, parameter estimates appear well-calibrated with a high correlation between the true and MLE values (Fig 5e–5h). While the coverage of our confidence intervals falls below the desired 95% level, we believe the coverage achieved is very reasonable given that the epidemic dynamics in the stochastic simulations can diverge considerably from what is expected under the pairwise model. In S1 Text, we in fact show that most of the estimation error can be attributed to stochastic variation in the epidemic dynamics (see S3 Fig).

### HIV-1 in Switzerland

**Fig 6:**
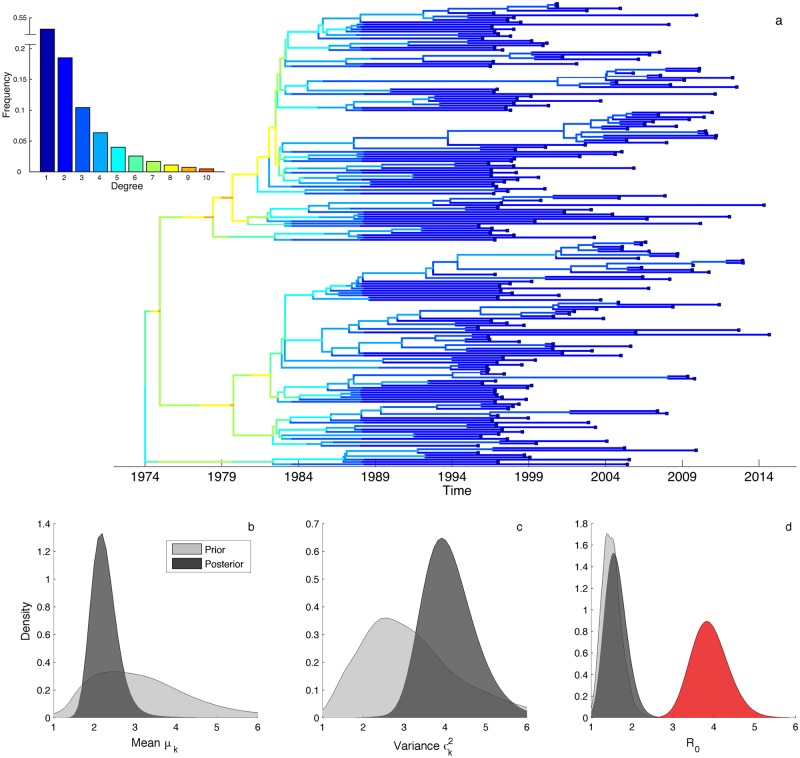
Phylodynamic analysis of a HIV-1 sub-epidemic among MSM in Switzerland. **(a)** Maximum clade credible phylogeny reconstructed from 200 HIV *pol* sequences. Lineages are colored by their expected degree in the network based on their ancestral degree distribution inferred under the pairwise coalescent model. The ancestral degree distribution of each lineage back through time was computed while conditioning on the median posterior estimate of all parameters. Inset shows the inferred degree distribution for the entire network (capped at *k* = 10 for visual simplicity). **(b-c)** Posterior (dark grey) and prior (light grey) distributions of the mean degree *μ*_*k*_ and variance $\sigma_{k}^{2}$ of the degree distribution. **(d)** Estimates of the basic reproductive number *R*_0_ inferred under the pairwise coalescent (grey) and under the random mixing coalescent model (red).

We performed a phylodynamic analysis of a HIV-1 subtype B epidemic among men-who-have-sex-with-men (MSM) in Switzerland using the pairwise coalescent model to see if we could estimate the statistical properties of a real-world sexual contact network. HIV *pol* sequences from infected individuals were obtained from patients enrolled in the Swiss HIV Cohort Study [47–49]. To minimize the effects of spatial structure, we focus on a single large sub-epidemic identified as primarily occurring in the Zürich region in a preliminary phylogenetic analysis (see S1 Text and S4 Fig). A time-calibrated phylogeny containing 200 sequences revealed this sub-epidemic to be quite genetically diverse with many older lineages originating in the early 1980’s (Fig 6a).

We fit a SIR-type pairwise epidemic model to this sub-epidemic while simultaneously inferring the tree from the sequence data in BEAST 2. We assumed a discretized gamma distribution for the degree distribution *d*_*k*_, which allowed us to independently estimate the mean *μ*_*k*_ and variance $\sigma_{k}^{2}$ of the network’s underlying degree distribution. The posterior estimates of *μ*_*k*_ and $\sigma_{k}^{2}$ indicate that the network was not especially well-connected (median *μ*_*k*_ = 2.20) but heterogenous in degree (median $\sigma_{k}^{2}=4.04$) (Fig 6b and 6c). The basic reproductive number was estimated to be between 1.0 and 2.5, although unlike for the network parameters the posterior density of *R*_0_ only diverged slightly from the prior (Fig 6d). *R*_0_ values estimated under the pairwise coalescent model were however significantly lower than the values estimated under the random mixing model, even though the same prior on *R*_0_ was used for both models.

Overall our phylodynamic analysis suggests that this particular sub-epidemic spread rapidly by way of a few highly connected individuals. This is supported by the inferred degree distribution of the network and can be seen from the expected degree of lineages computed from the inferred ancestral degree distribution of each lineage over time (Fig 6a). Most coalescent (i.e. transmission) events early in the epidemic are attributable to lineages residing in high degree individuals. Later, towards the beginning of the 2000’s, a few clusters in the tree begin to grow again through new transmission events along lineages with higher than average degree, which corresponds in time to the resurgence of HIV among MSM in Switzerland [48, 50].

## Discussion

Recent work has suggested that the structure of local contact networks can shape pathogen phylogenies [26–28]. Yet it remains unclear how much information pathogen phylogenies retain about the networks through which they spread and how to best extract information about network structure from trees. As a step towards addressing these questions, we sought a simple theoretical framework to explore the relationship between contact networks, epidemic dynamics, and phylogenies. Starting with random graph and pairwise epidemic models, we derived a fairly simple coalescent model that includes local network structure by using pair approximations. By treating the coalescent process as a backwards-time dynamical process on a network, our pairwise coalescent model allows us to capture the phylogenetic history of a pathogen in terms of how lineages move through a network and the rates at which they coalesce. As we have shown, our phylodynamic modeling framework provides a very good approximation to the coalescent process on random networks and can recapitulate the major features of pathogen phylogenies simulated on different types of random graphs.

Using the pairwise coalescent model and individual-based stochastic simulations as guides, we reexamined how contact network structure shapes pathogen phylogenies. We found that local contact network structure can have a strong impact on the the coalescent process in terms of the timing of coalescent events. Network properties like overall connectivity and contact heterogeneity that increase the epidemic growth rate concentrate coalescent events towards the beginning of an epidemic, while properties like clustering that slow epidemic growth broaden the distribution of coalescent events over the epidemic. On the other hand, properties like assortativity that were observed to have no strong effect on epidemic dynamics likewise have little influence on the timing of coalescent events (although we may have observed a larger effect if we had considered more extreme forms of assortative mixing [51–53]). This suggests that local contact network structure primarily shapes the coalescent process indirectly through the network’s influence on epidemic dynamics, particularly the timing of transmission events.

Because the pairwise coalescent model can be used for likelihood-based inference, it also offers a means of exploring how much information phylogenies contain about contact network structure. Using simulated phylogenies, our ability to infer network properties was highly dependent on the fraction of sampled individuals. While we could estimate network properties that strongly regulate epidemic dynamics such as overall connectivity even at sampling fractions as low as 10–25%, other properties that do not strongly regulate epidemic dynamics like assortativity proved difficult to precisely estimate even with complete sampling. This observation suggests that for the parameters that can be estimated at low sampling fractions, we may largely be inferring the structure of networks not from any direct signal of network structure in the tree itself, but from the indirect effect of network structure on the epidemic dynamics reflected in the timing of the coalescent events in the tree. Thus, without additional information beyond a phylogeny, network parameters may essentially be statistically unidentifiable from other epidemiological parameters like transmission rates that control epidemic dynamics.

Although it appears difficult to estimate some network properties from phylogenies, we were able to obtain informative estimates of the degree distribution of a sexual contact network underlying a large HIV sub-epidemic in Switzerland. While we were likely helped by the high fraction of HIV infected individuals sampled in Switzerland (at least 34–38%) compared with other countries and the relatively informative priors we placed on the model’s epidemiological parameters, this demonstrates that it is at least technically possible to estimate the structure of real-world contact networks from phylogenies. Our phylodynamic estimates indicated that the Swiss MSM network was not particularly well-connected with a rather low mean degree, but that the number of contacts per individual was highly variable. While a high degree of contact heterogeneity is consistent with other empirical studies of sexual contact networks [4, 6, 54], such low overall connectivity may be surprising in a high-risk population such as MSM. However, our estimates were based on a random graph model assuming a completely static network and therefore may better reflect the momentary degree distribution of the network in terms of the number of concurrent or nearly concurrent contacts individuals have rather than their total number of partners over time, which may grow substantially larger depending on the time they remain sexually active.

As in mathematical epidemiology, phylodynamics has made conceptual progress by developing simple models that largely assume random mixing [40, 55, 56]. It may now be worthwhile to consider when including network structure is likely to be important in phylodynamics. From earlier work in network epidemiology, it is well known that random mixing models can fail to provide an accurate description of epidemic dynamics because they ignore how local structure promotes or hinders transmission as well as how this local structure evolves over the course of an epidemic [14, 31, 57]. The timing of transmission events as regulated by local interactions on the network also appears to determine how well random mixing models can approximate the coalescent process on networks. On weakly connected or highly clustered networks where local interactions strongly limit transmission due to saturation effects, random mixing models overshoot the true transmission rate and therefore also the expected coalescent rate. In better connected networks, the effect of these local interactions on transmission is minimized by well-connected nodes and random mixing models can perform quite well [14]. The effect of local contact network structure on the coalescent process can therefore probably be safely ignored in highly connected networks, but may be important to consider in less well-connected networks. While network structure can also shape the topological characteristics of phylogenies such as tree imbalance, these effects appear to be quite weak [28], unless the distribution of contacts is extreme, as in the case of scale-free networks with power law degree distributions [27]. For multi-strain pathogen systems, network structure can have more complex effects on evolutionary dynamics depending on how different pathogens interact; ranging from a higher probability of invading strains being competitively excluded [58, 59] to facilitating the spread of synergistic co-infections [60]. The importance of multi-strain interactions on networks remains to be explored in phylodynamics.

Considering network structure may also be important when estimating and interpreting key epidemiological parameters. Of perhaps most interest to epidemiologists is *R*_0_, which is a function of both the transmissibility of a disease and contact network structure [8]. Thus, even if two pathogens are equally transmissible per contact, they can have substantially different *R*_0_ values based on contact patterns. Contact heterogeneity can for instance dramatically increase *R*_0_, while clustering can reduce *R*_0_ [61, 62]. But network structure can have an even more dramatic impact on epidemic dynamics, especially initial growth rates. For example, epidemics can spread very rapidly though heterogenous contact networks via a few highly connected individuals. In this case, *R*_0_ would likely be overestimated under a random mixing model because fast initial growth can only be explained under such a model by uniformly high transmission rates in the population; whereas in fact the highly connected individuals infected early in the epidemic do not represent the average connectedness of the population. This most likely explains why we estimated *R*_0_ for the Swiss HIV sub-epidemic to be more than twice as high under a random mixing model than under a model accounting for contact heterogeneity. Phylodynamic inference using random mixing models may be especially prone to large estimation errors in such settings because most phylogenetic information is concentrated in the early stages of an epidemic when most coalescent events occur just as network structure begins to shape epidemic dynamics.

While we strove for simplicity, the true complexity of real-world contact networks does highlight some deficiencies in the pairwise models. First, while we only considered perfectly static random graph models, real-world networks temporally evolve as new contacts form and dissolve. Pairwise epidemic models that allow for dynamic partner exchange have been proposed [63, 64], and in theory could be merged with our pairwise coalescent model to explore contact durations that are intermediate between the infinitesimal nature assumed by random mixing models and the permanent nature assumed by static models, as in [65]. Second, the deterministic pairwise models ignore the often considerable stochastic variability of epidemics on networks, which we found to be a major source of estimation error. Particle filtering approaches similar to those in [66] could be adapted to the pairwise coalescent model, although it is not straightforward to simply add stochasticity into pairwise models [67, 68]. Finally, the random graph models we employed here only consider local structure at the level of pairs in the network. Higher-order structure that subdivides networks into different communities most likely also plays a very strong role in shaping pathogen phylogenies. Developing methods that can quantify connectivity within and between communities while accounting for epidemic dynamics and incomplete sampling such as our approach does on local networks remains a challenging but highly important area of future research.

## Supporting information

S1 Text Supporting information on models, simulation methods and the HIV-1 phylogenetic analysis. Includes details on initial conditions for the pairwise epidemic model, how the pairwise coalescent model tracks lineage movement through networks, how epidemics and phylogenies were simulated, when the pairwise approximation fails due to higher-order community structure, how tree imbalance statistics were computed and normalized, how stochastic variability in epidemic dynamics can lead to estimation error under the deterministic pairwise models and details about the phylogenetic analysis of the Swiss HIV-1 sequence data.
(PDF)

S1 Fig The degree distribution of infected nodes and ancestral lineages over the time course of an epidemic. (TIF)

S2 Fig Accuracy of pairwise approximations on Watts-Strogatz networks with varying levels of higher-order community structure. (TIF)

S3 Fig The relationship between parameter estimation error and stochastic deviations in epidemic dynamics from those expected under the deterministic pairwise model. (TIF)

S4 Fig Size distribution of HIV sub-epidemics in Switzerland. (TIF)

S5 Fig Epidemiological and network parameters estimated from one phylogeny simulated to reflect the HIV epidemic in Switzerland. (TIF)

## References

[1] Anderson RM, May RM, Anderson B. Infectious diseases of humans: dynamics and control. vol. 28 Wiley Online Library; 1992.

[2] McCallum H, Barlow N, Hone J. How should pathogen transmission be modelled? Trends in Ecology and Evolution. 2001;16(6):295–300. 10.1016/S0169-5347(01)02144-9 11369107

[3] Read JM, Eames KT, Edmunds WJ. Dynamic social networks and the implications for the spread of infectious disease. Journal of The Royal Society Interface. 2008;5(26):1001–1007. 10.1098/rsif.2008.0013 PMC2607433 18319209

[4] Garnett GP, Hughes JP, Anderson RM, Stoner BP, Aral SO, Whittington WL, et al Sexual mixing patterns of patients attending sexually transmitted diseases clinics. Sexually Transmitted Diseases. 1996;23(3):248–257. 10.1097/00007435-199605000-00015 8724517

[5] Helleringer S, Kohler HP. Sexual network structure and the spread of HIV in Africa: evidence from Likoma Island, Malawi. AIDS. 2007;21(17):2323–2332. 10.1097/QAD.0b013e328285df98 18090281

[6] Liljeros F, Edling CR, Amaral LAN, Stanley HE, Åberg Y. The web of human sexual contacts. Nature. 2001;411(6840):907–908. 10.1038/35082140 11418846

[7] Rocha LE, Liljeros F, Holme P. Simulated epidemics in an empirical spatiotemporal network of 50,185 sexual contacts. PLoS Comput Biol. 2011;7(3):e1001109 10.1371/journal.pcbi.1001109 21445228 PMC3060161

[8] Meyers LA, Pourbohloul B, Newman ME, Skowronski DM, Brunham RC. Network theory and SARS: predicting outbreak diversity. Journal of Theoretical Biology. 2005;232(1):71–81. 10.1016/j.jtbi.2004.07.026 15498594 PMC7094100

[9] Faye O, Boëlle PY, Heleze E, Faye O, Loucoubar C, Magassouba N, et al Chains of transmission and control of Ebola virus disease in Conakry, Guinea, in 2014: an observational study. The Lancet Infectious Diseases. 2015;15(3):320–326. 10.1016/S1473-3099(14)71075-8 25619149 PMC4373532

[10] Scarpino SV, Iamarino A, Wells C, Yamin D, Ndeffo-Mbah M, Wenzel NS, et al Epidemiological and viral genomic sequence analysis of the 2014 ebola outbreak reveals clustered transmission. Clinical Infectious Diseases. 2015;60(7):1079–1082. 10.1093/cid/ciu1131 25516185 PMC4375398

[11] Newman ME. Spread of epidemic disease on networks. Physical Review E. 2002;66(1):016128 10.1103/PhysRevE.66.016128 12241447

[12] Pastor-Satorras R, Vespignani A. Immunization of complex networks. Physical Review E. 2002;65(3):036104 10.1103/PhysRevE.65.036104 11909162

[13] Keeling MJ, Eames KT. Networks and epidemic models. Journal of the Royal Society Interface. 2005;2(4):295–307. 10.1098/rsif.2005.0051 PMC1578276 16849187

[14] Bansal S, Grenfell BT, Meyers LA. When individual behaviour matters: homogeneous and network models in epidemiology. Journal of the Royal Society Interface. 2007;4(16):879–891. 10.1098/rsif.2007.1100 PMC2394553 17640863

[15] Liljeros F, Edling CR, Amaral LAN. Sexual networks: implications for the transmission of sexually transmitted infections. Microbes and Infection. 2003;5(2):189–196. 10.1016/S1286-4579(02)00058-8 12650777

[16] Eames K, Bansal S, Frost S, Riley S. Six challenges in measuring contact networks for use in modelling. Epidemics. 2015;10:72–77. 10.1016/j.epidem.2014.08.006 25843388

[17] Yerly S, Vora S, Rizzardi P, Chave JP, Vernazza P, Flepp M, et al Acute HIV infection: impact on the spread of HIV and transmission of drug resistance. AIDS. 2001;15(17):2287–2292. 10.1097/00002030-200111230-00010 11698702

[18] Ragonnet-Cronin M, Ofner-Agostini M, Merks H, Pilon R, Rekart M, Archibald CP, et al Longitudinal phylogenetic surveillance identifies distinct patterns of cluster dynamics. Journal of Acquired Immune Deficiency Syndromes. 2010;55(1):102–108. 10.1097/QAI.0b013e3181e8c7b0 20622676

[19] Brown AJL, Lycett SJ, Weinert L, Hughes GJ, Fearnhill E, Dunn DT. Transmission network parameters estimated from HIV sequences for a nationwide epidemic. Journal of Infectious Diseases. 2011;204(9):1463–1469. 10.1093/infdis/jir550 21921202 PMC3182313

[20] Leitner T, Escanilla D, Franzen C, Uhlen M, Albert J. Accurate reconstruction of a known HIV-1 transmission history by phylogenetic tree analysis. Proceedings of the National Academy of Sciences. 1996;93(20):10864–10869. 10.1073/pnas.93.20.10864 PMC38248 8855273

[21] Cottam EM, Thébaud G, Wadsworth J, Gloster J, Mansley L, Paton DJ, et al Integrating genetic and epidemiological data to determine transmission pathways of foot-and-mouth disease virus. Proceedings of the Royal Society of London B: Biological Sciences. 2008;275(1637):887–895. 10.1098/rspb.2007.1442 PMC2599933 18230598

[22] Jombart T, Eggo R, Dodd P, Balloux F. Reconstructing disease outbreaks from genetic data: a graph approach. Heredity. 2011;106(2):383–390. 10.1038/hdy.2010.78 20551981 PMC3183872

[23] Ypma R, Bataille A, Stegeman A, Koch G, Wallinga J, Van Ballegooijen W. Unravelling transmission trees of infectious diseases by combining genetic and epidemiological data. Proceedings of the Royal Society of London B: Biological Sciences. 2012;279(1728):444–450. 10.1098/rspb.2011.0913 PMC3234549 21733899

[24] Didelot X, Gardy J, Colijn C. Bayesian inference of infectious disease transmission from whole-genome sequence data. Molecular Biology and Evolution. 2014;31(7):1869–1879. 10.1093/molbev/msu121 24714079 PMC4069612

[25] Hall M, Woolhouse M, Rambaut A. Epidemic Reconstruction in a Phylogenetics Framework: Transmission Trees as Partitions of the Node Set. PLoS Comput Biol. 2015;11(12):e1004613 10.1371/journal.pcbi.1004613 26717515 PMC4701012

[26] O’Dea EB, Wilke CO. Contact heterogeneity and phylodynamics: how contact networks shape parasite evolutionary trees. Interdisciplinary Perspectives on Infectious Diseases. 2010;2011 10.1155/2011/238743 21151699 PMC2995904

[27] Leventhal GE, Kouyos R, Stadler T, Von Wyl V, Yerly S, Böni J, et al Inferring epidemic contact structure from phylogenetic trees. PLoS Comput Biol. 2012;8(3):e1002413–e1002413. 10.1371/journal.pcbi.1002413 22412361 PMC3297558

[28] Robinson K, Fyson N, Cohen T, Fraser C, Colijn C. How the dynamics and structure of sexual contact networks shape pathogen phylogenies. PLoS Comput Biol. 2013;9(6):e1003105 10.1371/journal.pcbi.1003105 23818840 PMC3688487

[29] Volz EM, Koopman JS, Ward MJ, Brown AL, Frost SD. Simple epidemiological dynamics explain phylogenetic clustering of HIV from patients with recent infection. PLoS Comput Biol. 2012;8(6):e1002552–e1002552. 10.1371/journal.pcbi.1002552 22761556 PMC3386305

[30] Newman M. Networks: An Introduction. Oxford University Press; 2010.

[31] Keeling MJ. The effects of local spatial structure on epidemiological invasions. Proceedings of the Royal Society of London B: Biological Sciences. 1999;266(1421):859–867. 10.1098/rspb.1999.0716 PMC1689913 10343409

[32] Rand D. Correlation equations and pair approximations for spatial ecologies. Advanced Ecological Theory: Principles and Applications. 1999;100 10.1002/9781444311501.ch4

[33] Eames KT, Keeling MJ. Modeling dynamic and network heterogeneities in the spread of sexually transmitted diseases. Proceedings of the National Academy of Sciences. 2002;99(20):13330–13335. 10.1073/pnas.202244299 PMC130633 12271127

[34] Bouckaert R, Heled J, Kühnert D, Vaughan T, Wu CH, Xie D, et al BEAST 2: a software platform for Bayesian evolutionary analysis. PLoS Comput Biol. 2014;10(4):e1003537 10.1371/journal.pcbi.1003537 24722319 PMC3985171

[35] Molloy M, Reed BA. A critical point for random graphs with a given degree sequence. Random Structures and Algorithms. 1995;6(2/3):161–180. 10.1002/rsa.3240060204

[36] Miller JC. Percolation and epidemics in random clustered networks. Physical Review E. 2009;80(2):020901 10.1103/PhysRevE.80.020901 19792067

[37] Newman ME. Random graphs with clustering. Physical Review Letters. 2009;103(5):058701 10.1103/PhysRevLett.103.058701 19792540

[38] Newman ME. Mixing patterns in networks. Physical Review E. 2003;67(2):026126 10.1103/PhysRevE.67.026126 12636767

[39] Taylor M, Simon PL, Green DM, House T, Kiss IZ. From Markovian to pairwise epidemic models and the performance of moment closure approximations. Journal of Mathematical Biology. 2012;64(6):1021–1042. 10.1007/s00285-011-0443-3 21671029

[40] Volz EM, Pond SLK, Ward MJ, Brown AJL, Frost SD. Phylodynamics of infectious disease epidemics. Genetics. 2009;183(4):1421–1430. 10.1534/genetics.109.106021 19797047 PMC2787429

[41] Frost SD, Volz EM. Viral phylodynamics and the search for an ‘effective number of infections’. Philosophical Transactions of the Royal Society B: Biological Sciences. 2010;365(1548):1879–1890. 10.1098/rstb.2010.0060 PMC2880113 20478883

[42] Koelle K, Rasmussen DA. Rates of coalescence for common epidemiological models at equilibrium. Journal of The Royal Society Interface. 2012;9(70):997–1007. 10.1098/rsif.2011.0495 PMC3306638 21920961

[43] Volz EM. Complex population dynamics and the coalescent under neutrality. Genetics. 2012;190(1):187–201. 10.1534/genetics.111.134627 22042576 PMC3249372

[44] House T, Keeling MJ. Insights from unifying modern approximations to infections on networks. Journal of The Royal Society Interface. 2011;8(54):67–73. 10.1098/rsif.2010.0179 PMC3024819 20538755

[45] Frost SD, Volz EM. Modelling tree shape and structure in viral phylodynamics. Philosophical Transactions of the Royal Society of London B: Biological Sciences. 2013;368(1614):20120208 10.1098/rstb.2012.0208 23382430 PMC3678332

[46] Blum MG, François O. On statistical tests of phylogenetic tree imbalance: the Sackin and other indices revisited. Mathematical biosciences. 2005;195(2):141–153. 10.1016/j.mbs.2005.03.003 15893336

[47] Ledergerber B, Egger M, Opravil M, Telenti A, Hirschel B, Battegay M, et al Clinical progression and virological failure on highly active antiretroviral therapy in HIV-1 patients: a prospective cohort study. The Lancet. 1999;353(9156):863–868. 10.1016/S0140-6736(99)01122-8 10093977

[48] Kouyos RD, Von Wyl V, Yerly S, Böni J, Taffé P, Shah C, et al Molecular epidemiology reveals long-term changes in HIV type 1 subtype B transmission in Switzerland. Journal of Infectious Diseases. 2010;201(10):1488–1497. 10.1086/651951 20384495

[49] Marzel A, Shilaih M, Yang WL, Böni J, Yerly S, Klimkait T, et al HIV-1 Transmission During Recent Infection and During Treatment Interruptions as Major Drivers of New Infections in the Swiss HIV Cohort Study. Clinical Infectious Diseases. 2016;62(1):115–122. 10.1093/cid/civ732 26387084

[50] Swiss Federal Office of Public Health. HIV und AIDS in der Schweiz. 2011;.

[51] Anderson R, Gupta S, Ng W. The significance of sexual partner contact networks for the transmission dynamics of HIV. JAIDS Journal of Acquired Immune Deficiency Syndromes. 1990;3(4):417–429. 2179528

[52] Newman ME. Assortative mixing in networks. Physical review letters. 2002;89(20):208701 10.1103/PhysRevLett.89.208701 12443515

[53] Badham J, Stocker R. The impact of network clustering and assortativity on epidemic behaviour. Theoretical Population Biology. 2010;77(1):71–75. 10.1016/j.tpb.2009.11.003 19948179

[54] Handcock MS, Jones JH. Likelihood-based inference for stochastic models of sexual network formation. Theoretical Population Biology. 2004;65(4):413–422. 10.1016/j.tpb.2003.09.006 15136015

[55] Pybus OG, Rambaut A, Harvey PH. An integrated framework for the inference of viral population history from reconstructed genealogies. Genetics. 2000;155(3):1429–1437. 10880500 10.1093/genetics/155.3.1429 PMC1461136

[56] Stadler T. On incomplete sampling under birth—death models and connections to the sampling-based coalescent. Journal of Theoretical Biology. 2009;261(1):58–66. 10.1016/j.jtbi.2009.07.018 19631666

[57] Barthélemy M, Barrat A, Pastor-Satorras R, Vespignani A. Dynamical patterns of epidemic outbreaks in complex heterogeneous networks. Journal of Theoretical Biology. 2005;235(2):275–288. 10.1016/j.jtbi.2005.01.011 15862595

[58] Ferdinandy B, Mones E, Vicsek T, Müller V. HIV competition dynamics over sexual networks: first comer advantage conserves founder effects. PLoS Comput Biol. 2015;11(2):e1004093 10.1371/journal.pcbi.1004093 25654450 PMC4318579

[59] Leventhal GE, Hill AL, Nowak MA, Bonhoeffer S. Evolution and emergence of infectious diseases in theoretical and real-world networks. Nature communications. 2015;6 10.1038/ncomms7101 25592476 PMC4335509

[60] Hébert-Dufresne L, Althouse BM. Complex dynamics of synergistic coinfections on realistically clustered networks. Proceedings of the National Academy of Sciences. 2015;112(33):10551–10556. 10.1073/pnas.1507820112 PMC4547298 26195773

[61] Keeling M. The implications of network structure for epidemic dynamics. Theoretical Population Biology. 2005;67(1):1–8. 10.1016/j.tpb.2004.08.002 15649519

[62] Volz EM, Miller JC, Galvani A, Meyers LA. Effects of heterogeneous and clustered contact patterns on infectious disease dynamics. PLoS Comput Biol. 2011;7(6):e1002042 10.1371/journal.pcbi.1002042 21673864 PMC3107246

[63] Bauch C, Rand D. A moment closure model for sexually transmitted disease transmission through a concurrent partnership network. Proceedings of the Royal Society of London B: Biological Sciences. 2000;267(1456):2019–2027. 10.1098/rspb.2000.1244 PMC1690763 11075716

[64] Eames KT, Keeling MJ. Monogamous networks and the spread of sexually transmitted diseases. Mathematical Biosciences. 2004;189(2):115–130. 10.1016/j.mbs.2004.02.003 15094315

[65] Volz E, Meyers LA. Susceptible—infected—recovered epidemics in dynamic contact networks. Proceedings of the Royal Society of London B: Biological Sciences. 2007;274(1628):2925–2934. 10.1098/rspb.2007.1159 PMC2291166 17878137

[66] Rasmussen DA, Volz EM, Koelle K. Phylodynamic inference for structured epidemiological models. PLoS Comput Biol. 2014;10(4):e1003570 10.1371/journal.pcbi.1003570 24743590 PMC3990497

[67] Dangerfield C, Ross JV, Keeling MJ. Integrating stochasticity and network structure into an epidemic model. Journal of the Royal Society Interface. 2009;6(38):761–774. 10.1098/rsif.2008.0410 PMC2586797 18974032

[68] Graham M, House T. Dynamics of stochastic epidemics on heterogeneous networks. Journal of Mathematical Biology. 2014;68(7):1583–1605. 10.1007/s00285-013-0679-1 23633042

