# Supplementary material for: Phylodynamics on local sexual contact networks
Source: PLoS Comput Biol. 2017 Mar 28;13(3):e1005448. doi: 10.1371/journal.pcbi.1005448 (PMC5388502; doi:10.1371/journal.pcbi.1005448)
Supplement: S1 Text — Includes details on initial conditions for the pairwise epidemic model, how the pairwise coalescent model tracks lineage movement through networks, how epidemics and phylogenies were simulated, when the pairwise approximation fails due to higher-order community structure, how tree imbalance statistics were computed and normalized, how stochastic variability in epidemic dynamics can lead to estimation error under the deterministic pairwise models and details about the phylogenetic analysis of the Swiss HIV-1 sequence data. (PDF) [file pcbi.1005448.s001.pdf]

Phylodynamics on local sexual contact networks

David A. Rasmussen[1,2*], Roger Kouyos[3,4], Huldrych F. Günthard[3,4], Tanja Stadler[1,2]

1 Department of Biosystems Science and Engineering, ETH Zürich, Basel, Switzerland
2 Swiss Institute of Bioinformatics, Lausanne, Switzerland
3 Division of Infectious Diseases and Hospital Epidemiology, University Hospital Zürich, University of Zürich, Zürich, Switzerland
4 Institute of Medical Virology, University of Zürich, Zürich, Switzerland

* david.rasmussen@bsse.ethz.ch

S1 Text

Initial conditions for the pairwise epidemic model

The initial conditions for the pairwise epidemic model depend on the statistical properties of the network described by a random graph model, including its degree distribution d_k and edge degree distribution e_{kl}. At time $t = 0$, we assume a single individual becomes infected from outside of the network such that $[I]^{init} = \sum_k [I_k]^{init} = 1$. The total number of susceptible individuals is therefore $[S]^{init} = \sum_k [S_k]^{init} = N - [I]^{init}$, where N is the total number of nodes in the network. To be consistent with the random graph model, the degree distribution of the susceptible and infected population must reflect the overall degree distribution d_k:

$$\begin{aligned} S_k^{init} &= p_k S^{init} \\ I_k^{init} &= p_k I^{init}. \end{aligned} \tag{1}$$

At the level of pairs, we assume that the initially infected individual has an average number of contacts given by the mean degree of the network μ_k. Thus, the initial number of SI pairs $[SI]^{init} = \sum_k \sum_l [S_k I_l] = \mu_k$. We assume there are initially no II pairs, so $[SS]^{init} = \sum_k \sum_l [S_k S_l] = P - \mu_k$. The total number of pairs in the network $P = \frac{\mu_k N}{2}$. To be consistent with the random graph model, the degree distribution over each type of pair must reflect the edge degree distribution e_{kl}:

$$\begin{aligned} S_k S_l^{init} &= e_{kl} SS^{init} \\ S_k I_l^{init} &= e_{kl} SI^{init} \\ I_k I_l^{init} &= 0. \end{aligned} \tag{2}$$

For random graph models with assortative mixing, e_{kl} can be parameterized to reflect correlations in the degree of connected nodes. Otherwise, if pairs are assumed to form randomly between nodes independently of their degree:

$$e_{kl} = \frac{k d_k l d_l}{\mu_k^2}. \tag{3}$$

Tracking lineage movement on networks

Here we consider how lineages move through a network in terms of the ancestral degree distribution of a lineage. Going backwards in time, the degree of a lineage will transition from k to l whenever the lineage is transmitted from a degree l individual to a degree k individual in forward time. Transitions from k to l in backwards time are written as $l \leftarrow k$ so that the direction of time is transparent. With incomplete sampling, a lineage may be transmitted between two nodes at a coalescent event that went unobserved in the tree because the parent lineage was not sampled. A lineage will therefore transition between states along branches in the tree each time an unobserved transmission event occurs between nodes of unequal degree. Thus, the rate at which $l \leftarrow k$ transitions occur along a lineage currently in state k is equal to the rate at which the lineage coalesces with lineages in state l (through a $l \rightarrow k$ transmission event) that are not among the sampled lineages in the phylogeny. Assuming for the moment that there are no lineages in the phylogeny currently in state l, the rate at which $l \leftarrow k$ transitions occur along a branch is

$$\gamma^{l \leftarrow k} = \frac{\tau [S_k I_l] \chi_{kl} [I_l]}{[I_k I_l]}, \tag{4}$$

which is the rate of coalescence between a lineage in state k and all $[I_l]$ lineages in the population.

Notice that if $k \neq l$, then $\chi_{kl} = [I_k I_l]/[I_k]][I_l]$, Eq (4) simplifies to

$$\gamma^{l \leftarrow k} = \frac{\tau [S_k I_l]}{[I_k]}, \tag{5}$$

which has the more intuitive interpretation that a lineage transitions from state k to l at the same rate at which $l \rightarrow k$ transmission events occur in the population multiplied by the probability $\frac{1}{I_k}$ that it is this particular lineage in state k that is transmitted.

If the phylogeny contains lineages in state l, we need to consider that in order for the coalescent event to appear as a $l \leftarrow k$ transition along a branch, the parent lineage must not be among the sampled lineages in the phylogeny. As suggested by [1], the expected number of sampled lineages a_l in state l can be approximated from the lineage state probabilities as $a_l \approx \sum_i p_{il}$. We can then substitute the $[I_l]$ term in Eq (4) with the probable number of lineages in state l but not in the phylogeny: $[I_l] - a_l$.

Given these transition rates, we can write down master equations for how p_{ik} changes backwards in time:

$$\frac{d}{dt} p_{ik} = \sum_l \left(\gamma^{k \leftarrow l} p_{il} - \gamma^{l \leftarrow k} p_{ik} \right). \tag{6}$$

These master equations allow us to compute the probability of a lineage being in a given state at any time in the past, which we refer to as the ancestral degree distribution of a lineage. However, since we generally do not know the degree of sampled individuals, we need to place a prior on p_{ik} at the time of sampling. We use the degree distribution of the infected population at the time of sampling as a natural prior on the initial values of p_{ik}:

$$p_{ik}^{init} = \frac{I_k}{\sum_k I_k}. \tag{7}$$

To obtain the degree distribution of the infected population, we can numerically solve the ODEs for I_k under the pairwise epidemic model.

Finally, we need to consider how the lineage state probabilities get updated after an observed coalescent event in the tree. Specifically, we need to compute the state probabilities for the parent lineage h after its daughter lineages i and j coalesce. This is:

$$p_{hk} = \frac{1}{\lambda_{ij}} \sum_{l} \frac{\tau[S_l I_k]\chi_{kl}}{[I_k I_l]} \mathcal{P}_{kl}, \tag{8}$$

which is just the normalized probability of the parent being in state k conditional on $\mathcal{P}_{kl}$ and the rate at which transmission events occur.

In order to see if the master equations given in Eq (6) provide an accurate representation of how lineages move through a network, we compare our theoretical expectations of p_k with stochastic simulations where we recorded the state of a single sampled lineage backwards through time in each realization. In the population at large, well-connected nodes with high degree are overrepresented in the infected population early in an epidemic but the degree distribution of infected nodes rapidly converges to a stationary and approximately uniform distribution where all nodes have an equal probability of being infected regardless of degree (Fig S 1a). Relative to the infected population at large, the ancestral degree distribution reconstructed from IBS simulations reveals that sampled lineages have an even higher probability of being in well-connected nodes during the early stages of an epidemic (Fig S 1b). This results from lineages in higher degree nodes leaving more descendants and therefore having a higher probability of being ancestral to a sampled lineage. The master equations used by the pairwise coalescent model to track lineage movement reproduce this pattern almost exactly, although there is some disagreement during the earliest stages of the epidemic when $I_k << 1$ for all k (Fig S 1c).

Simulation methods

For each individual-based stochastic (IBS) simulation, we first generated a random network with the desired statistical properties using the configuration model [2]. If the network was not completely connected with all nodes connected to all others by at least one path in the network, it was discarded and a new one generated. To seed the epidemic, a single node was then randomly chosen to become infected at time $t = 0$. The simulations then proceeded forwards in time using an event-driven approach similar to the Gillespie stochastic simulation algorithm [3]. Infected hosts were allowed to either transmit to their susceptible neighbors or recover from infection. At transmission events, the parent and child pathogen lineage were recorded so that the ancestry of each lineage could be traced in order to recover the true phylogeny of the pathogen population. At recovery events, infected individuals were sampled with probability ρ and subsequently included in the phylogeny. At the final time $t = T$, all surviving infections were also sampled with probability ρ and included in the phylogeny. Unless otherwise stated, infected individuals were sampled serially though time upon removal (i.e. recovery).

Phylogenies were simulated under the pairwise coalescent model backwards in time. At time $t = T$, sampled individuals were added to a set of lineages that were then traced back through time. The degree of sampled lineages was drawn randomly from the degree distribution of the infected population at the time of sampling according to the pairwise epidemic model. The state of each lineage was then updated incrementally using small time steps receding into the past. At each discrete time step, lineages could either transition to a different degree node or coalesce with another lineage with probabilities proportional the rate of degree transitions given in Eq (4) above and the coalescent rate given in the main text. Simulations were run until the final two ancestral lineages coalesced.

To measure the error in the theoretical expectations provided by the pairwise models when compared against individual-based stochastic (IBS) simulations on networks, we

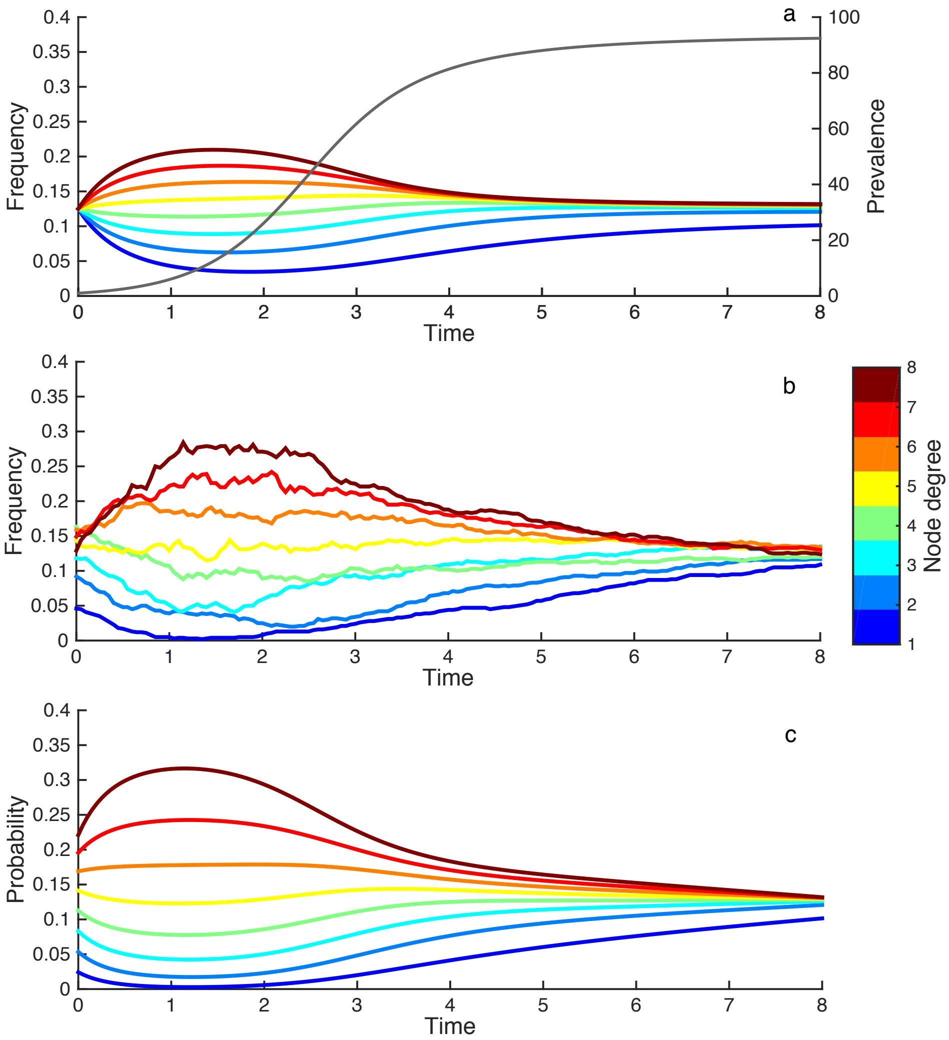

Fig S 1. The degree distribution of infected nodes and ancestral lineages over the time course of an epidemic. (a) Degree distribution for the entire infected population. At time $t = 0$ we start with a uniform degree distribution for the initial infected host that mirrors the uniform degree distribution of the underlying network. The overlaid grey line represents prevalence over time. **(b)** The ancestral degree distribution for a single lineage traced backwards through time from 1000 stochastic simulations **(c)** Theoretical expectation for the ancestral degree of a single lineage given by the master equations derived from the pairwise model.

approximate the time-integrated mean error $\bar{E}$ in prevalence and coalescent distributions by averaging over all T time points on a discretized interval, such that

$$\bar{E} = \frac{1}{T}\sum_{i=1}^{T} |z_{pw}(t_i) - z_{stoch}(t_i)|, \tag{9}$$

where z_{pw} is the value given by the pairwise model and z_{stoch} is the mean value given by the stochastic simulations.

For IBS simulations used to generate phylogenies to test our inference methods, the mean error $\bar{E}$ in the number of infected individuals over time between IBS simulations and the dynamics expected under the pairwise epidemic model was capped at 10% of the network size N. Simulations exceeding this threshold were discarded to partially minimize the effects of stochastic epidemic dynamics on inference.

When the pairwise approximation fails

From the general theory of dynamical processes on networks, we expect pair approximations to work well when dynamical correlations arise locally at the level of pairs or other lower-order motifs like triples, but may break down when there is significant higher-order network structure, such as when the network is modular or broken up into different communities [4, 5]. Given that the coalescent process can also be viewed as a dynamical process on a network (albeit backwards in time), we expect that the pair approximations underlying the pairwise coalescent will also break down in the presence of higher-order network structure. To explore how higher-order structure affects the accuracy of the pairwise models, we used the well-known Watts-Strogatz model [6] to generate networks with varying levels of higher-order structure.

To simulate random graphs under the Watt-Strogatz model we start with an initially perfectly ordered ring network where each node is connected to its $\hat{k}$ nearest neighbors and then randomly rewire a fraction of edges f. A low f therefore preserves the original community structure present in the ring whereas a high f randomizes the network in a way that destroys higher-order structure (Fig S 2). Our variant of this algorithm uses degree-preserving rewiring so that we can study the effects of community structure without introducing additional contact heterogeneity.

The time-integrated mean error in the theoretical expectations provided by the pairwise models for both prevalence and the distribution of coalescent events is shown in Fig S 2. The error arising from the pair approximation is only large when the rewiring fraction is very low ($f <= 0.10$) and there is substantial community structure in the networks. Moreover, the pairwise coalescent model appears to break down at the same point as the pairwise epidemic model, which is not surprising given that the coalescent model depends on the accuracy of the epidemic dynamics predicted by the pairwise epidemic model. While networks with $f <= 0.10$ have high clustering coefficients, this does not appear to be the ultimate downfall of the pairwise models because there is already substantial clustering with $f > 0.10$ where the pairwise models still perform well (Fig S 2). Rather, where the pairwise models break down at $f <= 0.10$ is also the point at which we see a large spike in the mean internode distance, the minimum distance between two nodes in a network (Fig S 2).

Thus, the pairwise models perform well as long as the networks are sufficiently "small" as quantified by mean internode distances, which will rise sharply once the network is broken up into different communities. This echoes an earlier observation made by [5], who showed low-dimensional models that ignore higher-order community structure can provide surprisingly accurate approximations to dynamics on a variety of complex networks as long as networks are sufficiently small-world.

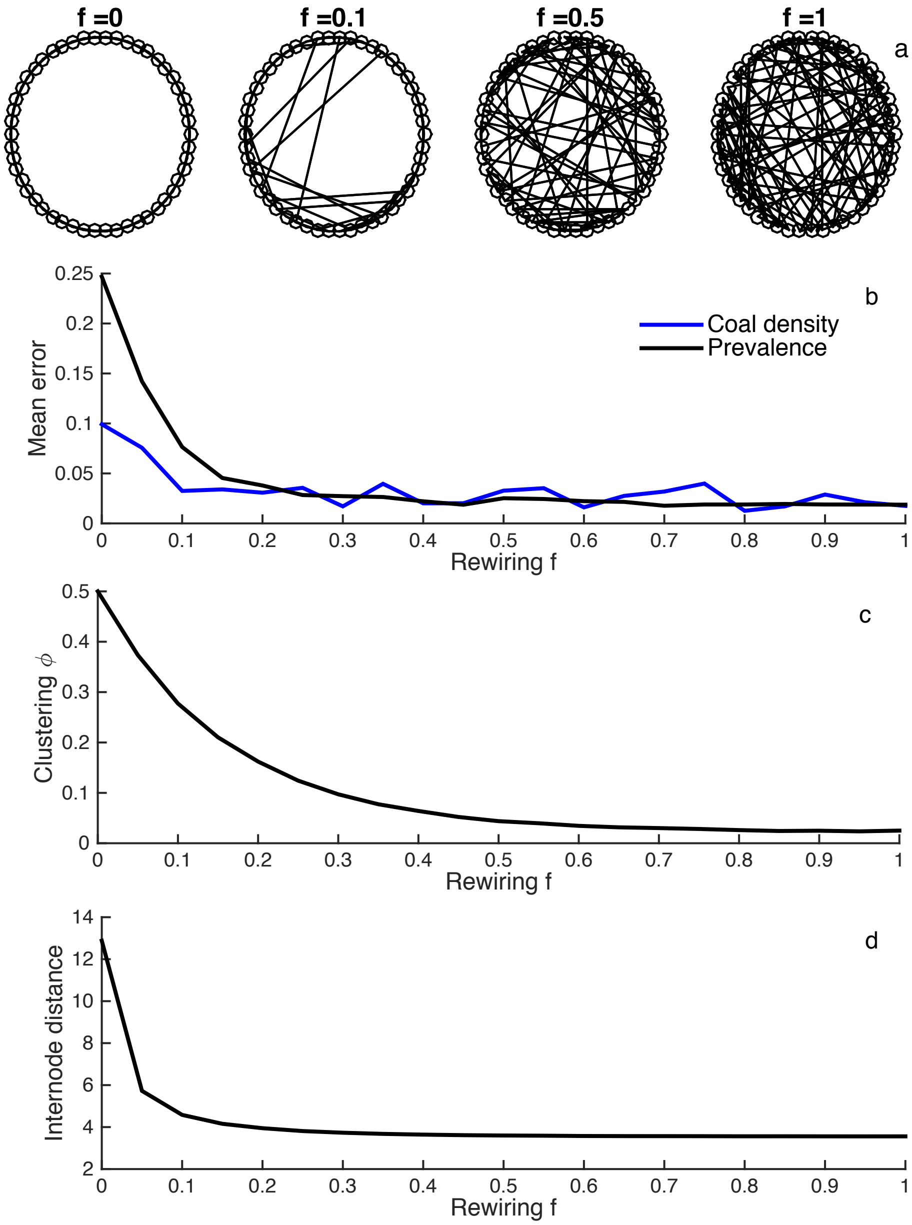

Fig S 2. Accuracy of pairwise approximations on Watts-Strogatz networks with varying levels of higher-order community structure. (a) Watts-Strogatz networks with different edge rewiring fractions f. For ease of viewing, $N = 50$ here. **(b)** Time-integrated mean error in the coalescent density (blue) and prevalence (black) given by the pairwise models when compared against stochastic simulations on randomly generated networks with different f values. For each f value, 500 stochastic simulations were performed to compute the mean error in the pairwise approximation. **(c)** The mean clustering coefficient ϕ of simulated networks for each f value. **(d)** The mean internode distance for the same networks as in b and c. For all simulations $\hat{k} = 4$ and $N = 250$.

Measuring phylogenetic tree imbalance

We used three different measures of phylogenetic tree shape to quantify how tree imbalance increases with network contact heterogeneity in trees generated using both individual-based stochastic simulations and backward-time simulations of the pairwise coalescent model.

Colless's index I_c considers the asymmetry in the number of samples descending from the children of each internal node u [7]. Letting T_u^L and T_u^R be the number of samples descending from the left and right children of u, then

$$I_c(n) = \frac{2}{(n-1)(n-2)} \sum_u ||T_u^L - T_u^R||, \tag{10}$$

where n is the total number of sampled lineages [8, 9].

As shown by [8], if the number of samples n is even, the expected value of the index $\mathbb{E}(I_c(n))$ is:

$$\mathbb{E}(I_c(n)) = \frac{2n}{(n-1)(n-2)} \sum_{j=2}^{n/2} \frac{1}{j}. \tag{11}$$

If n is odd,

$$\mathbb{E}(I_c(n)) = \frac{2n}{(n-1)(n-2)} \left[\frac{1}{n} + \sum_{j=2}^{(n-1)/2} \frac{1}{j} \right]. \tag{12}$$

Sackin's index I_s considers the distance d_v between each sampled tip in the tree v and the root in terms of the number of internal nodes that need to be traversed [10, 11]:

$$I_s = \sum_v d_v. \tag{13}$$

The expected value of Sackin's index $\mathbb{E}(I_s(n)) \approx 2n \log(n)$ under the Yule model for large n [12].

We also consider the number of cherries C – or sample pairs that share an immediately adjacent common ancestor in the tree. The expected number of cherries $\mathbb{E}(C(n)) = \frac{n}{3}$ under the Yule model [13].

Following [14], for each imbalance measure $I(n)$ we compute a normalized value

$$\bar{I}(n) = \frac{I(n) - \mathbb{E}(I(n))}{\mathbb{E}(I(n))}, \tag{14}$$

based on the expected value of the statistic for a Yule tree with the same number of samples in order to adjust for different sample sizes between trees.

The effect of stochastic epidemic dynamics on parameter estimates

In the main text, we showed that the pairwise coalescent model is able to provide reliable and generally accurate estimates of network parameters. However, for some stochastic simulations the error in estimated parameter values was large and coverage was lower than expected. One potential source of this error is stochastic variation in epidemic dynamics in the IBS simulations, which can deviate widely from the mean dynamics expected under the pairwise models. Because the deterministic pairwise models cannot account for this variability, errors in parameter estimates may result.

In order to see how much error in the parameter estimates could be attributed to stochasticity in the epidemic dynamics, we simulated an additional set of trees where we tracked the stochastic deviation in the number of infected individuals in the IBS

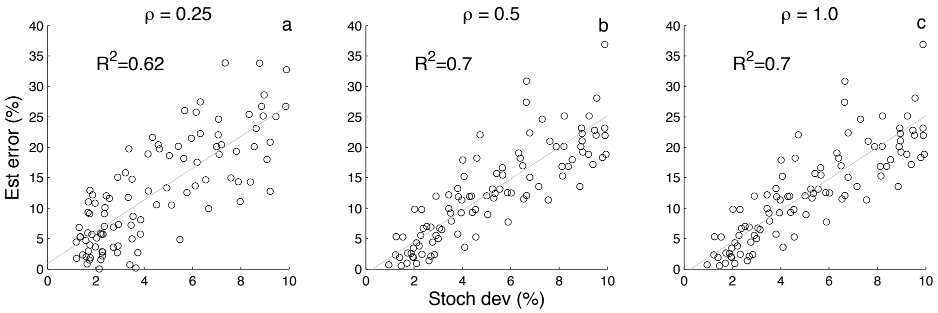

Fig S 3. Error in the connectivity parameter estimated from simulated trees regressed on the stochastic deviation of the epidemic dynamics from those expected under the deterministic pairwise model. The estimation error is given in terms of the percentage deviation away from the true value used in simulations. The stochastic deviation in the epidemic dynamics is given in terms of the time-integrated mean error $\bar{E}$ in the percentage of individuals infected expected under the deterministic pairwise model relative to the IBS simulation. The least-squares regression line is shown in grey along with the resulting coefficient of determination R^2. The regression is shown at three different sampling fractions: (a) $\rho = 0.25$, (b) $\rho = 0.5$, and (c) $\rho = 1.0$.

simulations from the expected mean dynamics. We then estimated the connectivity parameter μ_k from each stochastic simulation. Fig S 3 shows the error in the ML parameter estimates regressed on the stochastic deviation of each simulation from the mean dynamics. The estimation error is highly correlated with the size of the stochastic deviation and the R^2 values suggest that between 60 - 70% of the variation in estimates can be explained by stochasticity. Increasing the sampling fraction ρ does not appear to alter the relationship between the estimation error and stochastic deviations nor reduce the amount of overall error.

Phylogenetic analysis of Swiss HIV-1 sequence data

HIV-1 subtype B *pol* sequences were obtained from the Swiss HIV Cohort Study (SHCS). The SHCS includes a representative fraction of all HIV infected individuals in Switzerland (hereafter CH) and includes 45-50% of all cases ever diagnosed in CH [15, 16]. Viral sequences have been sampled since 1995, but sampling has intensified since 2000 when routine genotypic drug resistance testing began [15]. Currently, the sequence database contains samples from 60% of the over 19,000 enrolled patients [17] and at least 75% of patients enrolled since 1996. Therefore, approximately 34-38% of all cases ever diagnosed in CH are included in the sequence database, although this estimate is based on the entire HIV epidemic in CH and coverage is likely to be higher for more recent years and specific risk groups like men-who-have-sex-with-men (MSM) [18]. For an exhaustive description of the representativeness of the SHCS and the respective sequence data, see the supplementary information in [16]. While the Swiss epidemic includes a mixture of population risk groups including heterosexuals, injection drug users and MSM, we only analyzed viral samples from MSM.

The HIV-1 epidemic in CH is strongly integrated into the general European subtype B epidemic, especially among MSM [15, 19]. We therefore first tried to identify sub-epidemics primarily occurring on local contact networks within CH rather than abroad. We combined 4441 subtype B *pol* sequences taken from MSM patients enrolled in the (SHCS) with a large background dataset of 4550 subtype B sequences from the Los Alamos National Laboratory (LANL) HIV database. After removing all

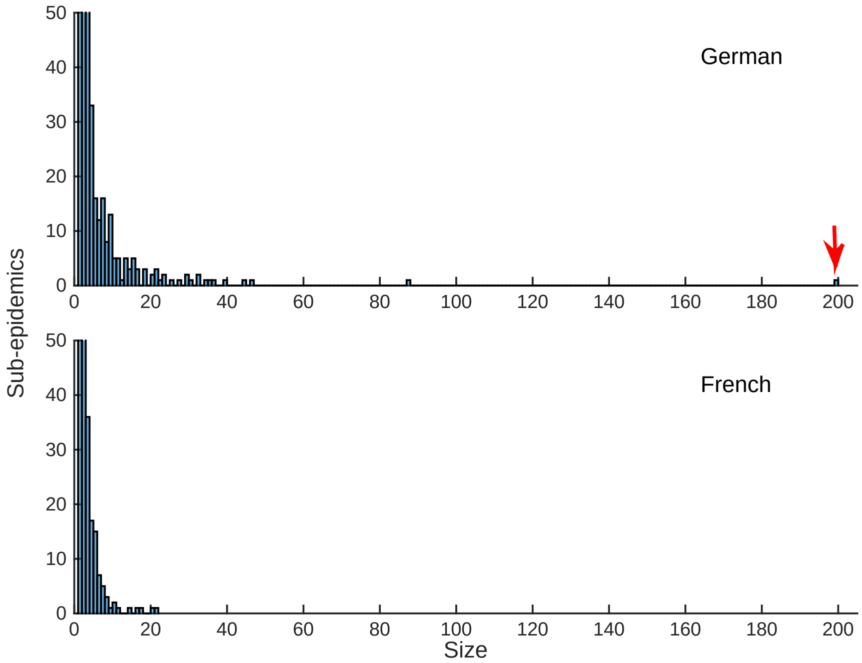

Fig S 4. Size distribution of HIV sub-epidemics in Switzerland. Sub-epidemics were categorized as occurring in either the German or French speaking regions of Switzerland. Size refers to the number of sampled individuals included in each sub-epidemic. The red arrow marks the large sup-epidemic we chose to analyze in detail.

non-subtype B and recombinant sequences, the SHCS and LANL sequences were then aligned together against the HXB2 subtype B reference strain. After alignment, a total of 51 codon positions associated with known drug resistance mutations were also stripped from the alignment. A maximum likelihood (ML) phylogeny of the combined LANL + SHCS alignment was then reconstructed in FastTree [20] assuming a GTR model of molecular evolution with gamma distributed rate heterogeneity.

To identify sub-epidemics occurring predominantly within CH, we first reconstructed the ancestral location of all internal nodes using maximum parsimony. Introductions into CH were assumed to occur whenever a node inferred to be in CH had a parent node outside of CH. Sub-epidemics were then defined to include all lineages sampled in CH that descended from an introduction event into CH without passing through a node reconstructed to be outside of CH. This preliminary analysis revealed that the Swiss epidemic is composed of many sub-epidemics likely originating from independent introductions into CH. Most of these sub-epidemics are composed of only a few sampled individuals and can be categorized as occurring predominantly in either the French or German speaking region of CH (Fig S 4). To further minimize the effects of geographic structure within CH on our phylodynamic analysis, we chose to focus on a large cluster which included 200 sampled individuals who predominantly lived or sought treatment in the Zürich area.

Using the 200 sequences from this sub-epidemic, we estimated the underlying network's degree distribution together with the time-calibrated viral phylogeny using BEAST 2 [21]. For phylogenetic reconstruction, we assumed a strict molecular clock

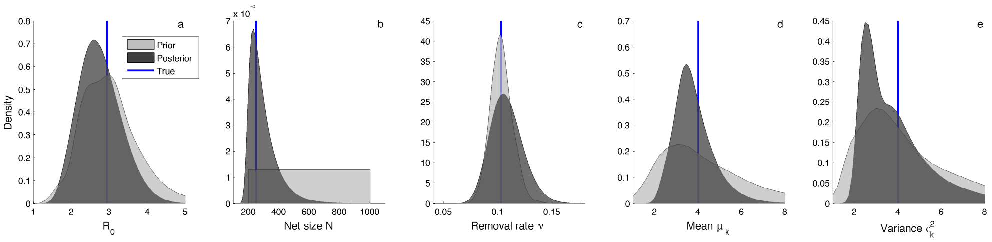

Fig S 5. Epidemiological and network parameters estimated from one representative simulated HIV phylogeny. Posterior (dark grey) and prior (light grey) distributions of all parameters are shown relative to the true values (blue) used in the simulation. Note that slightly different priors were used to validate the method than for the analysis of the empirical Swiss HIV data shown in Table S 1.

and a GTR model of nucleotide substitution. A SIR-type pairwise coalescent model was used as a tree prior, which allowed us to jointly estimate the posterior distribution of trees together with the parameters controlling the networks degree distribution. The degree distribution was assumed to follow a discretized gamma distribution. In general, we used fairly informative priors on the epidemiological parameters in the model but relatively uninformative priors on the network parameters (Table S 1). Posterior distributions for all model parameters and the tree were inferred using BEAST's built-in MCMC sampling algorithm. The pairwise coalescent model was implemented in BEAST 2 as an add-on package called PairTree, freely available at https://github.com/davidrasm/PairTree.

Before analyzing the empirical HIV sequences, we first tested the implementation of the pairwise coalescent in BEAST and its ability to accurately estimate both epidemiological and network parameters from mock phylogenies simulated to reflect the Swiss HIV epidemic. By placing informative priors on R_0 and the removal rate ν, we were able to accurately estimate the mean μ_k and variance σ_k^2 of the degree distribution as well as the overall size of the contact network N. The posterior versus prior distribution of all estimated parameters is shown in Fig S 5 for one representative simulation. Additional mock HIV phylogenies used to validate the inference method are available in BEAST XML input files alongside of the source code.

Table S 1. Priors on the parameters used in the Swiss HIV analysis.

Parameter	Prior distribution
R_0	LogNormal(0.4055, 0.15)
Removal rate ν	LogNormal(-2.28, 0.1)
Net size N	Uniform(200, 1000)
Mean μ_k	LogNormal(1.0986, 0.4)
Variance σ_k^2	LogNormal(1.0986, 0.4)

Parameters for the LogNormal distribution are the (log) mean and variance. For the Uniform distribution, the lower and upper limit.

References

1. Volz EM. Complex population dynamics and the coalescent under neutrality. Genetics. 2012;190(1):187–201.

2. Molloy M, Reed BA. A critical point for random graphs with a given degree sequence. Random Structures and Algorithms. 1995;6(2/3):161–180.
3. Gillespie DT. Stochastic simulation of chemical kinetics. Annu Rev Phys Chem. 2007;58:35–55.
4. Taylor M, Simon PL, Green DM, House T, Kiss IZ. From Markovian to pairwise epidemic models and the performance of moment closure approximations. Journal of Mathematical Biology. 2012;64(6):1021–1042.
5. Melnik S, Hackett A, Porter MA, Mucha PJ, Gleeson JP. The unreasonable effectiveness of tree-based theory for networks with clustering. Physical Review E. 2011;83(3):036112.
6. Watts DJ, Strogatz SH. Collective dynamics of 'small-world' networks. Nature. 1998;393(6684):440–442.
7. Colless DH. Review of phylogenetics: the theory and practice of phylogenetic systematics. Syst Zool. 1982;31:100–104.
8. Heard SB. Patterns in tree balance among cladistic, phenetic, and randomly generated phylogenetic trees. Evolution. 1992; p. 1818–1826.
9. Robinson K, Fyson N, Cohen T, Fraser C, Colijn C. How the dynamics and structure of sexual contact networks shape pathogen phylogenies. PLoS Comput Biol. 2013;9(6):e1003105.
10. Sackin M. "Good" and "bad" phenograms. Systematic Biology. 1972;21(2):225–226.
11. Shao KT. Tree balance. Systematic Biology. 1990;39(3):266–276.
12. Kirkpatrick M, Slatkin M. Searching for evolutionary patterns in the shape of a phylogenetic tree. Evolution. 1993; p. 1171–1181.
13. McKenzie A, Steel M. Distributions of cherries for two models of trees. Mathematical biosciences. 2000;164(1):81–92.
14. Leventhal GE, Kouyos R, Stadler T, Von Wyl V, Yerly S, Böni J, et al. Inferring epidemic contact structure from phylogenetic trees. PLoS Comput Biol. 2012;8(3):e1002413–e1002413.
15. Kouyos RD, Von Wyl V, Yerly S, Böni J, Taffé P, Shah C, et al. Molecular epidemiology reveals long-term changes in HIV type 1 subtype B transmission in Switzerland. Journal of Infectious Diseases. 2010;201(10):1488–1497.
16. Yang WL, Kouyos R, Scherrer AU, Böni J, Shah C, Yerly S, et al. Assessing the paradox between transmitted and acquired HIV type 1 drug resistance mutations in the Swiss HIV Cohort Study from 1998 to 2012. Journal of Infectious Diseases. 2015;212(1):28–38.
17. Marzel A, Shilaih M, Yang WL, Böni J, Yerly S, Klimkait T, et al. HIV-1 Transmission During Recent Infection and During Treatment Interruptions as Major Drivers of New Infections in the Swiss HIV Cohort Study. Clinical Infectious Diseases. 2016;62(1):115–122.

18. Drescher SM, von Wyl V, Yang WL, Böni J, Yerly S, Shah C, et al. Treatment-naive individuals are the major source of transmitted HIV-1 drug resistance in men who have sex with men in the Swiss HIV Cohort Study. Clinical infectious diseases. 2014;58(2):285–294.

19. Paraskevis D, Pybus O, Magiorkinis G, Hatzakis A, Wensing AM, Vijver DA, et al. Tracing the HIV-1 subtype B mobility in Europe: a phylogeographic approach. Retrovirology. 2009;6(1):1.

20. Price MN, Dehal PS, Arkin AP. FastTree 2–approximately maximum-likelihood trees for large alignments. PloS One. 2010;5(3):e9490.

21. Bouckaert R, Heled J, Kühnert D, Vaughan T, Wu CH, Xie D, et al. BEAST 2: a software platform for Bayesian evolutionary analysis. PLoS Comput Biol. 2014;10(4):e1003537.
