# Supplementary figures and images for: Phylodynamics on local sexual contact networks
Source: PLoS Comput Biol. 2017 Mar 28;13(3):e1005448. doi: 10.1371/journal.pcbi.1005448 (PMC5388502; doi:10.1371/journal.pcbi.1005448)

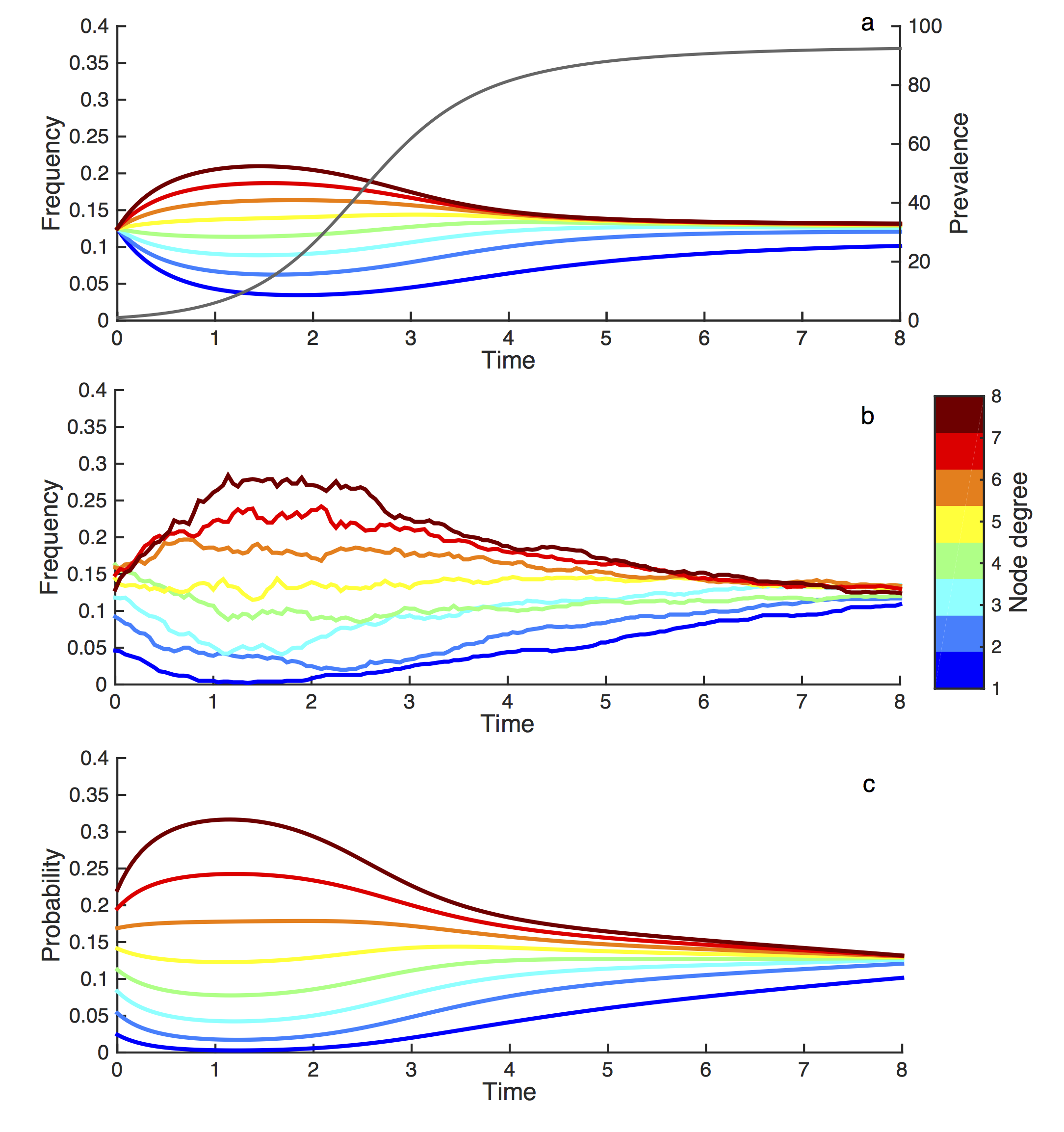

Supplement: S1 Fig — (TIF) [file pcbi.1005448.s002.tif]

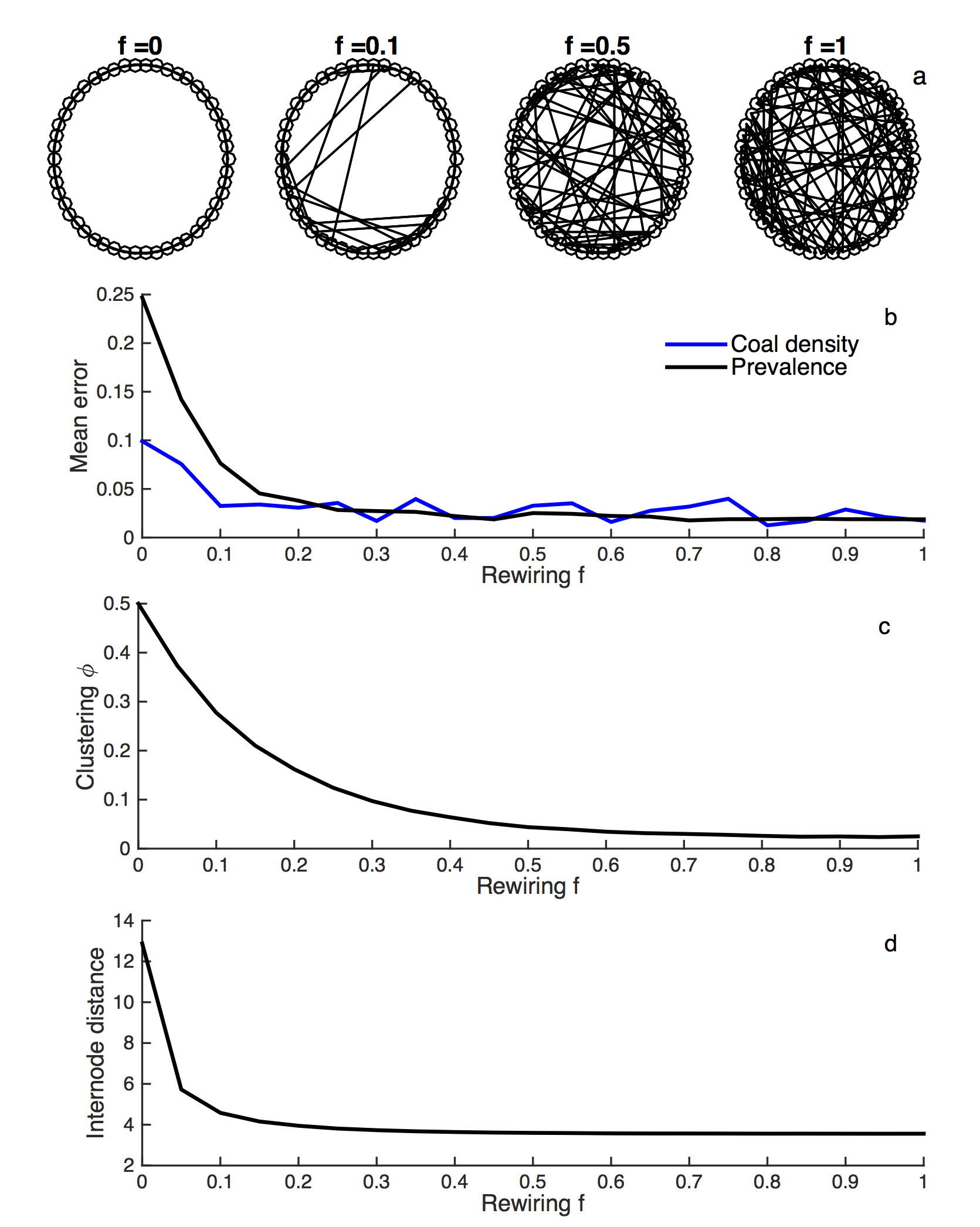

Supplement: S2 Fig — (TIF) [file pcbi.1005448.s003.tif]

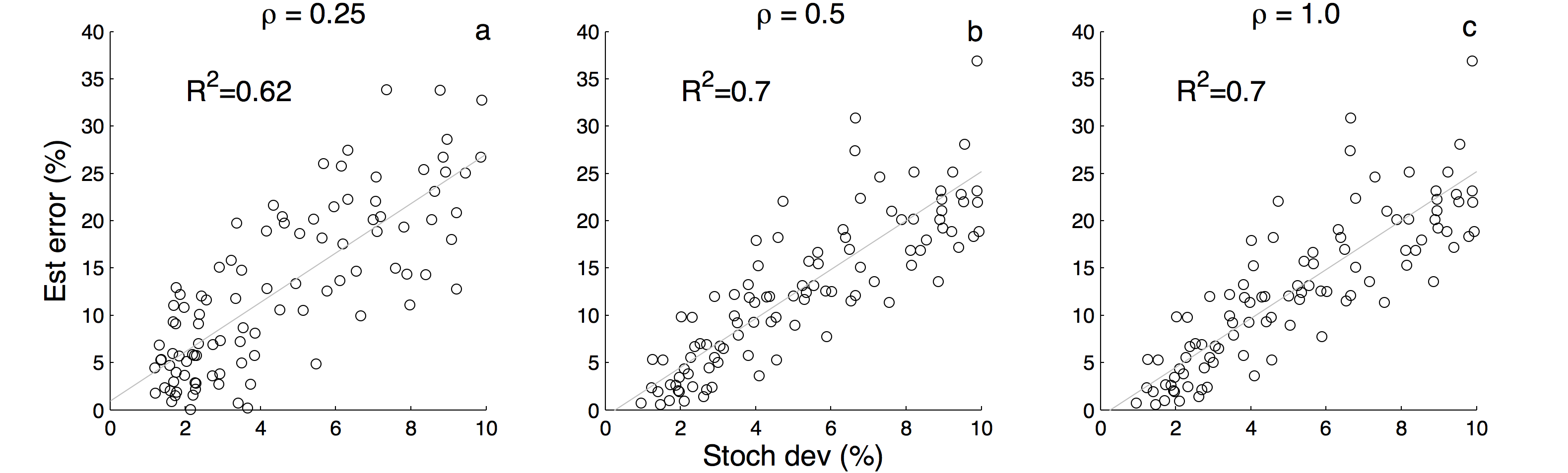

Supplement: S3 Fig — (TIF) [file pcbi.1005448.s004.tif]

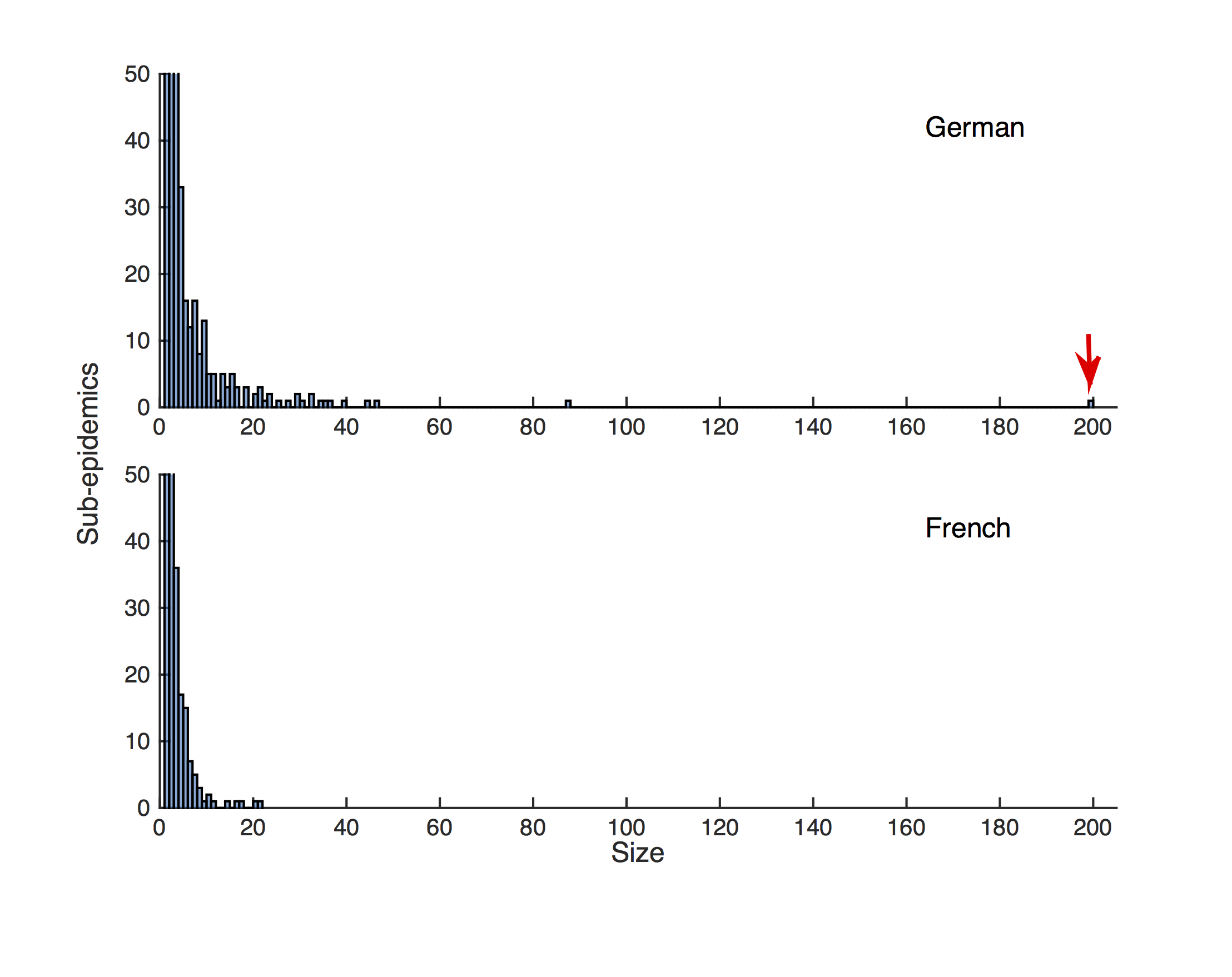

Supplement: S4 Fig — (TIF) [file pcbi.1005448.s005.tif]

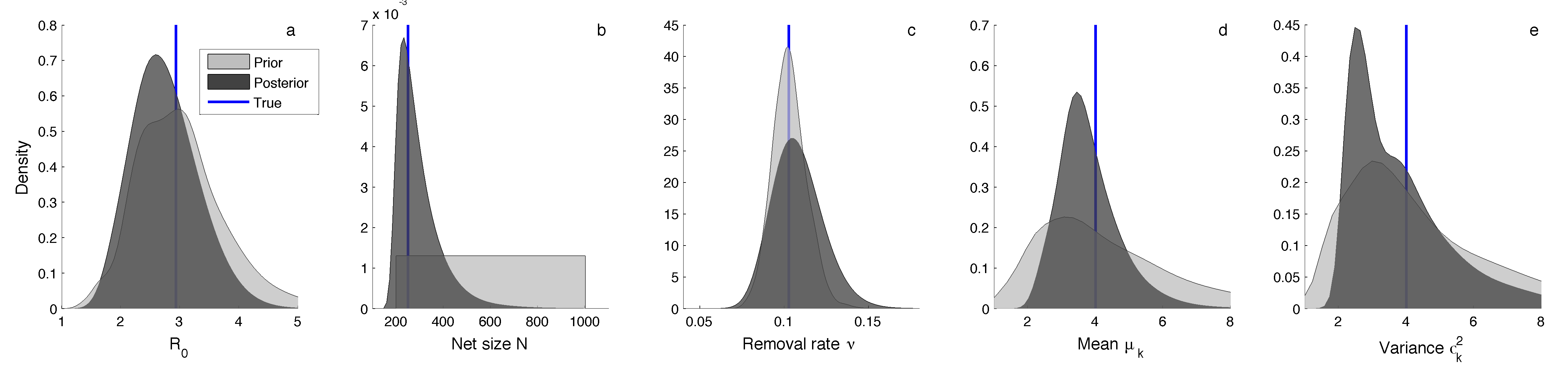

Supplement: S5 Fig — (TIF) [file pcbi.1005448.s006.tif]
